# The *CaCIPK3* gene positively regulates drought tolerance in pepper

**DOI:** 10.1038/s41438-021-00651-7

**Published:** 2021-10-01

**Authors:** Xiao Ma, Yang Li, Wen-Xian Gai, Chuang Li, Zhen-Hui Gong

**Affiliations:** College of Horticulture, Northwest A&F University, Yangling, Shaanxi 712100 P. R. China

**Keywords:** Drought, Plant signalling

## Abstract

Drought stress is a major agricultural problem restricting the growth, development, and productivity of plants. Calcineurin B-like proteins (CBLs) and CBL-interacting protein kinases (CIPKs) significantly influence the plant response to different stresses. However, the molecular mechanisms of CBL–CIPK in the drought stress response of pepper are still unknown. Here, the function of *CaCIPK3* in the regulation of drought stress in pepper (*Capsicum annuum* L.) was explored. Transcriptomic data and quantitative real-time PCR (qRT-PCR) analysis revealed that *CaCIPK3* participates in the response to multiple stresses. Knockdown of *CaCIPK3* in pepper increased the sensitivity to mannitol and methyl jasmonate (MeJA). Transient overexpression of *CaCIPK3* improved drought tolerance by enhancing the activities of the antioxidant system and positively regulating jasmonate (JA)-related genes. Ectopic expression of *CaCIPK3* in tomato also improved drought and MeJA resistance. As the CaCIPK3-interacting partner, CaCBL2 positively influenced drought resistance. Additionally, CaWRKY1 and CaWRKY41 directly bound the *CaCIPK3* promoter to influence its expression. This study shows that *CaCIPK3* acts as a positive regulator in drought stress resistance via the CBL–CIPK network to regulate MeJA signaling and the antioxidant defense system.

## Introduction

Pepper (*Capsicum annuum* L.) is an economically important horticultural plant belonging to the Solanaceae family. Pepper fruits are rich in vitamins, pigments, and pungent compounds and are widely used in various cuisines and chemical industries^1^. The harvested area and production of pepper have increased significantly in recent years. The latest FAO data show that the annual production of pepper was 38.03 million tons worldwide in 2019^2^.

Plants need to evolve complex mechanisms against diverse stresses under current global environmental deterioration^3^. In the northwest region of China, drought is a critical stress and significantly affects plant production. Exploring the molecular mechanisms of plants under drought stress can help enhance drought tolerance. Molecules such as calcium (Ca^2+^), reactive oxygen species (ROS), and abscisic acid (ABA) act as long-distance messengers in the regulation of drought stress^4^. Additionally, methyl jasmonate (MeJA) induces stomatal closure in response to drought stress^5,6^.

Ca^2+^ functions as an important second messenger and is related to various signaling pathways involved in drought stress^7^. The intracellular Ca^2+^ concentration is rapidly increased under abiotic and biotic stresses. The changes are captured by Ca^2+^ sensors, which transduce Ca^2+^ signals to downstream target proteins. As a special type of Ca^2+^ sensor, CBLs interact with their partner CBL-interacting protein kinases (CIPKs) to phosphorylate various substrates^8^. Recently, the functions of the CBL–CIPK network were extensively explored^9^. The typical CBL–CIPK network is a salt overly sensitive (SOS) pathway related to salt stress and was first identified in *Arabidopsis*. *SOS1* (plasma membrane-localized Na^+^/H^+^ exchanger 7), *SOS3* (*AtCBL4*), and *SOS2* (*AtCIPK24*) coregulate ion homeostasis to enhance salt tolerance by phosphorylation^10–12^. AtCIPK24 and the homologous member AtCIPK8 interact with AtCBL10 to activate Na^+^ extrusion^13^. In addition, the regulation of Na^+^, nitrate (NO_3_^−^), potassium (K^+^), and ABA signaling is influenced by the AtCBL1/AtCBL9-AtCIPK23 complex with different target proteins^14–17^. Compared with other well-known CBL–CIPK signaling pathways, studies related to drought and MeJA stress in Solanaceae vegetables are scarce.

Drought is currently the most severe environmental stress because of climate change and global warming^18^. Drought stress triggers ROS and ABA production, induces stomatal closure, and activates specific genes associated with signal transduction pathways^19,20^. In *Arabidopsis*, overexpression of *AtCBL1* or *AtCBL5* enhances drought tolerance^21,22^. In contrast, *AtCIPK11* overexpression enhances sensitivity to drought stress^23^. The rice gene *OsCIPK23* and wheat genes *TaCIPK2* and *TaCIPK27* positively regulate drought stress^24–26^. Transgenic apple-overexpressing *MdCIPK22* was shown to exhibit higher sugar production and drought resistance based on MdSUT2.2^27^. In grapevine, *VaCIPK02* positively influenced drought stress via ABA signaling and ROS accumulation^28^.

MeJA is a common phytohormone that participates in defense responses, plant development, and secondary metabolite biosynthesis^29^. Coronatine-insensitive 1 (COI1), jasmonate ZIM-domain (JAZ), and myelocytomatosis (MYC) proteins are the core members of the JA signaling pathway^30,31^. Many transcription factors, such as MYC, NAC, MYB, and WRKY, participate in the JA signaling pathway^30,32^. Furthermore, JA induces the pheophorbide *α* oxygenase (PAO)/phyllobilin pathway during senescence. The accumulation of pheide *α* activates JA-responsive genes^30,33^. Previous studies show that some promoters of *CIPKs*, including *AtCIPK6* and *VaCIPK02*, contain the TGACG motif (involved in MeJA responsiveness)^28,34^. Several *BrrCBLs* and *BrrCIPKs* are induced by MeJA treatment^35^. However, the functions of CBLs and CIPKs in response to drought and MeJA have not been revealed in pepper.

Our previous study revealed that *CaCIPK3* is influenced by salt and osmotic stress^36^. In the present study, the role of *CaCIPK3* in the regulation of drought and MeJA resistance was explored. Knocking down *CaCIPK3* expression decreased drought and MeJA tolerance in pepper. In contrast, drought and MeJA resistance increased in *CaCIPK3-*overexpressing plants. Drought stress activated the antioxidant system to scavenge ROS in *CaCIPK3-*overexpressing plants. Moreover, the expression of JA-related genes was altered. CaCBL2, the interacting partner of CaCIPK3, resulted in a drought-sensitive phenotype in *CaCBL2*-silenced pepper. Furthermore, the activity of the *CaCIPK3* promoter was regulated by CaWRKY1 and CaWRKY41. Collectively, these findings show that *CaCIPK3* potentially coregulates CaCBL2 and CaWRKYs via MeJA signaling and the antioxidant defense system to enhance drought tolerance in pepper.

## Results

### Sequence, subcellular localization, and evolutionary analyses of CaCIPK3

CIPKs are named based on their order on the chromosome^36^. *CaCIPK3* is the third gene and located on chromosome 1. *CaCIPK3* encodes a protein with 440 amino acid residues and a molecular weight of 49.81 kDa. *CaCIPK3* belongs to an intron-poor clade that contains zero introns. MSA analysis showed that CaCIPK3 exhibits a typical structure of the CIPK family. The NAF motif is located between the protein kinase domain and the PPI motif. In addition, CaCIPK3 harbors transmembrane helices, suggesting that it may be localized at the membrane (Supplementary Fig. S1A). To explore the position and potential function of *CaCIPK3*, the gene was fused between the CaMV35S promoter and GFP (Supplementary Fig. S1B). The construct (pvbg2307:CaCIPK3-GFP) was transiently expressed in *Nicotiana tabacum*. Expression of the control (pvbg2307:GFP) was observed throughout the cell, whereas the CaCIPK3–GFP protein was only visible in the plasma membrane. A phylogenetic analysis was performed to analyze the evolutionary relationship of CIPKs in many species. According to the results, CaCIPK3 was most similar to tomato SlCIPK14 and tobacco NtCIPK14 (Supplementary Fig. S1C).

### Expression analysis of *CaCIPK3* in pepper and *Arabidopsis*

**Fig. 1:**
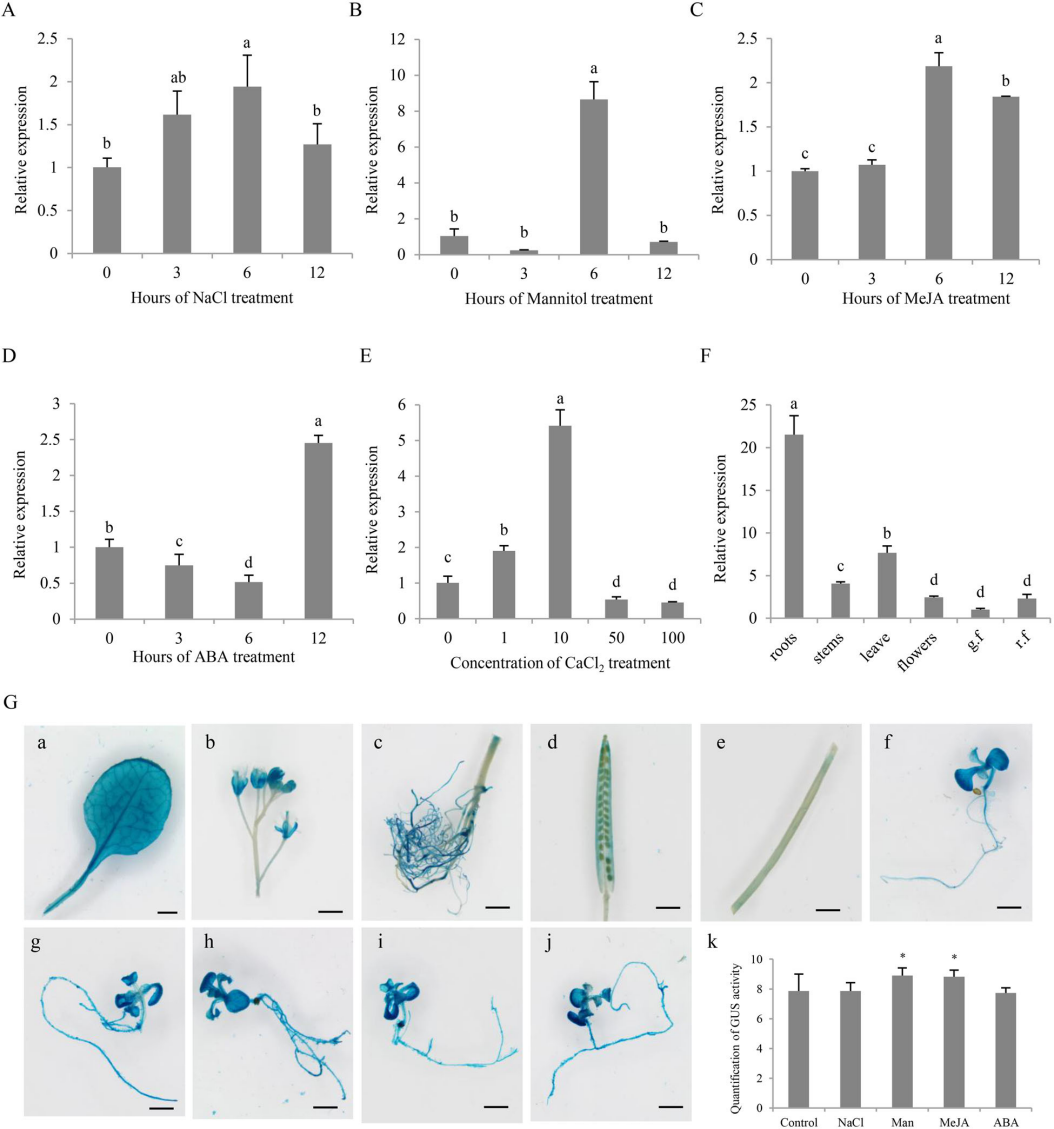
Expression profile of *CaCIPK3* in plants. **A**, **B***CaCIPK3* expression in pepper roots after treatment with NaCl and mannitol. **C**, **D** and **E** *CaCIPK3* expression in pepper leaves after treatment with MeJA, ABA, and CaCl_2_. **F** Tissue-specific expression of *CaCIPK3* in pepper. The values are the means ± SE (standard error) of three independent replicates. The letters (a–d) indicate significant differences according to Tukey’s test (*p* < 0.05). **G** GUS staining of *CaCIPK3pro:GUS* transgenic plants in leaves (**a**), flowers (**b**), roots (**c**), seeds (**d**), and stems (**e**). GUS staining in seedlings of *CaCIPK3pro:GUS* transgenic plants in response to sterile water (**f**), 0.15 M NaCl (**g**), 0.3 M mannitol (**h**), 0.1 mM MeJA (**i**), and 2 µM ABA (**j**). (**k**) Quantification of GUS activity in response to different stresses (*, *p* < 0.05). Scale bars, 1 mm

The expression patterns of *CaCIPK3* under various stresses and hormones were integrated with published RNA-seq data (http://pepperhub.hzau.edu.cn/) to explore its potential functions^37^. *CaCIPK3* responded to multiple stresses, including mannitol, NaCl, ABA, JA, cold, and heat (Supplementary Fig. S1D). Quantitative RT-PCR was performed to further verify the transcriptomic data of *CaCIPK3* expression under NaCl, mannitol, MeJA, and ABA treatments at different time points (Fig. 1A–D). *CaCIPK3* expression was slightly increased after NaCl and MeJA treatment, and the expression was the highest at 6 h posttreatment. ABA application decreased *CaCIPK3* expression before 6 h but enhanced its expression at 12 h. Under mannitol treatment, *CaCIPK3* expression peaked at the 6-h time point. Altogether, these results demonstrate that mannitol, MeJA, and ABA induce *CaCIPK3* expression. Considering that CaCIPK3 is a member of the Ca^2+^ signaling pathway, we assessed whether Ca^2+^ influenced *CaCIPK3* expression. The expression of *CaCIPK3* was examined under different concentrations of CaCl_2_ at the 6-h time point. *CaCIPK3* expression was induced by lower CaCl_2_ concentrations and downregulated by higher concentrations (>50 mM) (Fig. 1E), implying that *CaCIPK3* may be vital in the Ca^2+^ signaling pathway.

*CaCIPK3* transcript levels were assessed in different tissues of pepper to understand its spatial expression. The *CaCIPK3* transcript levels were the highest in roots and leaves (Fig. 1F). Spatial expression was also affected by the promoter. A total of 1500 bp of the *CaCIPK3* promoter was selected to analyze *cis*-acting elements. We found four MeJA-related *cis*-acting elements (Supplementary Table S1), implying that *CaCIPK3* may participate in MeJA signaling. Notably, a W-box (TTGACC) element, which is potentially recognized by WRKY proteins, was identified in the promoter, indicating that WRKY proteins might regulate *CaCIPK3*. *Arabidopsis* was transformed with the pCAMBIA-*CaCIPK3*_*pro*_*:GUS* vector to detect whether the *CaCIPK3* promoter influences GUS expression in different tissues. The GUS activity of *CaCIPK3*_*pro*_ was higher in the leaves, flowers, and roots than in the seeds and stems of mature plants (Fig. 1G a–e). In two-week-old *Arabidopsis* seedlings, *CaCIPK3* was expressed in all organs, especially in cotyledons. Notably, GUS activity in the fresh leaves of seedlings was induced by mannitol and MeJA but not salt or ABA (Fig. 1G f–k).

### Silencing of *CaCIPK3* decreases pepper resistance to drought and MeJA

**Fig. 2:**
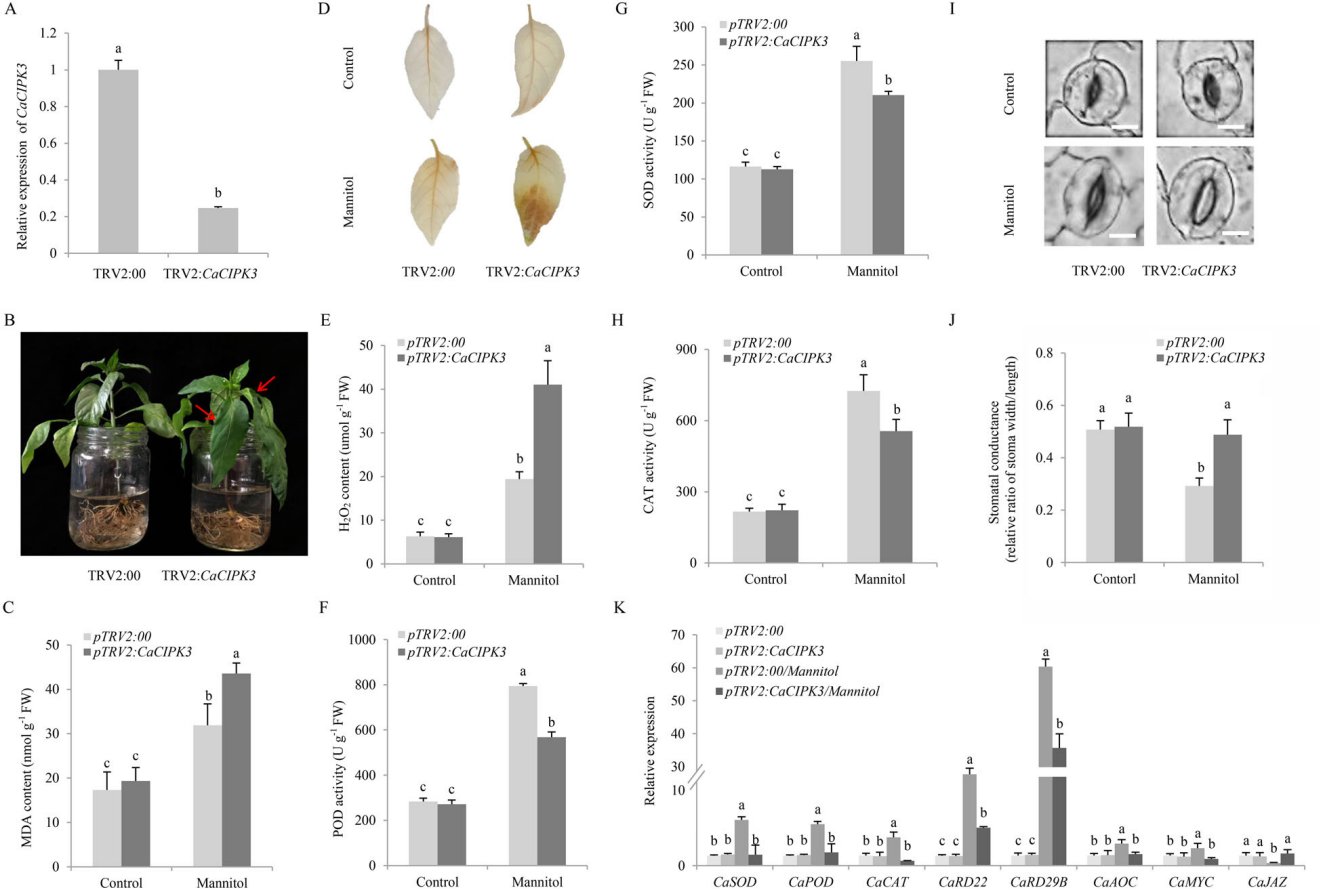
Knockdown of *CaCIPK*3 decreases mannitol resistance in pepper. **A** The expression level of *CaCIPK3* in *CaCIPK3-*silenced and control plants. **B** Appearance of pepper exposed to 300 mM mannitol. **C** MDA content. **D** H_2_O_2_ staining. **E** H_2_O_2_ content. **F** POD activity. **G** SOD activity. **H** CAT activity. **I**, **J** Stomatal aperture analysis. Scale bar, 10 µm. **K** The expression levels of *CaSOD*, *CaPOD*, *CaCAT*, *CaRD22*, *CaRD29B*, *CaAOC*, *CaMYC*, and *CaJAZ* in *CaCIPK3*-silenced and control plants under normal conditions and mannitol treatments. The values represent the means of three independent replicates ± SE (standard error). The letters (**a**–**c**) show significant differences according to Tukey’s test (*p* < 0.05)

Excess mannitol and MeJA activated *CaCIPK3* expression, implying that *CaCIPK3* potentially participates in drought and MeJA stress responses. A VIGS assay was performed to examine the effect of *CaCIPK3* silencing in pepper under mannitol-induced dehydration stress^38^ and MeJA treatments. A specific 309-bp fragment of *CaCIPK3* was selected to silence *CaCIPK3* in pepper. Four weeks after injection, *CaCIPK3* expression significantly decreased, confirming that *CaCIPK3* was successfully silenced in the leaves (Fig. 2A). After four days of hydroponic cultivation, the plants were immersed in 300 mM mannitol to simulate drought stress. Compared with control plants, the *CaCIPK3*-silenced leaves significantly wilted at 6 h post treatment (Fig. 2B). The leaves of *CaCIPK3*-silenced plants accumulated more MDA, which reflected the degree of membrane damage (Fig. 2C). H_2_O_2_ accumulation in the control plants and *CaCIPK3*-silenced plants was determined using DAB staining and further quantified by detection kits (Solarbio, China). The *CaCIPK3*-silenced plants exhibited more H_2_O_2_ accumulation than the control plants (Fig. 2D–E). Furthermore, the members of the antioxidant system that scavenge excess ROS, including POD, SOD, and CAT, were measured. These enzymes were remarkably increased in the control plants under mannitol stress (Fig. 2F–H). Stomatal morphology was examined after 6 h of mannitol stress in the control and *CaCIPK3*-silenced plants. No significant difference in the stomatal apertures (width-to-length ratio) was observed between the control and *CaCIPK3*-silenced plants under normal conditions. However, the stomata shrank under mannitol treatment, but the stomatal apertures were decreased in the control plants (Fig. 2I, J). We also detected the expression of antioxidant-related genes (*CaSOD*, *CaPOD*, and *CaCAT*) in the control versus *CaCIPK3*-silenced plants. The expression levels of these genes were higher in the leaves of control plants than in the leaves of *CaCIPK3*-silenced plants (Fig. 2F). Dehydration-related genes (*CaRD22* and *CaRD29B*) were rapidly induced in the leaves of both *CaCIPK3*-silenced and control plants under mannitol treatment. The expression of these genes was higher in the leaves of control plants (Fig. 2F). The expression of JA biosynthesis and signaling pathway genes (*CaAOC*, *CaMYC*, and *CaJAZ*) was altered in *CaCIPK3*-silenced and control plants. Compared with the leaves of *CaCIPK3*-silenced plants, the expression of *CaAOC* and *CaMYC* was higher in the control leaves, whereas the transcript level of *CaJAZ* was lower (Fig. 2F).

Leaf disks from control and *CaCIPK3*-silenced leaves were immersed in 400 µM MeJA solutions or sterile water under normal conditions to explore the role of *CaCIPK3* in MeJA. After seven days, the *CaCIPK3*-silenced leaf disks were more chlorotic and yellow than the controls (Supplementary Fig. S2A). The chlorophyll contents were significantly reduced in *CaCIPK3*-silenced plants compared with control plants (Supplementary Fig. S2A). Collectively, the results suggest that *CaCIPK3* is critical for mannitol-induced drought stress and MeJA tolerance in pepper.

### Transient overexpression of *CaCIPK3* enhanced tolerance to drought and MeJA in pepper

**Fig. 3:**
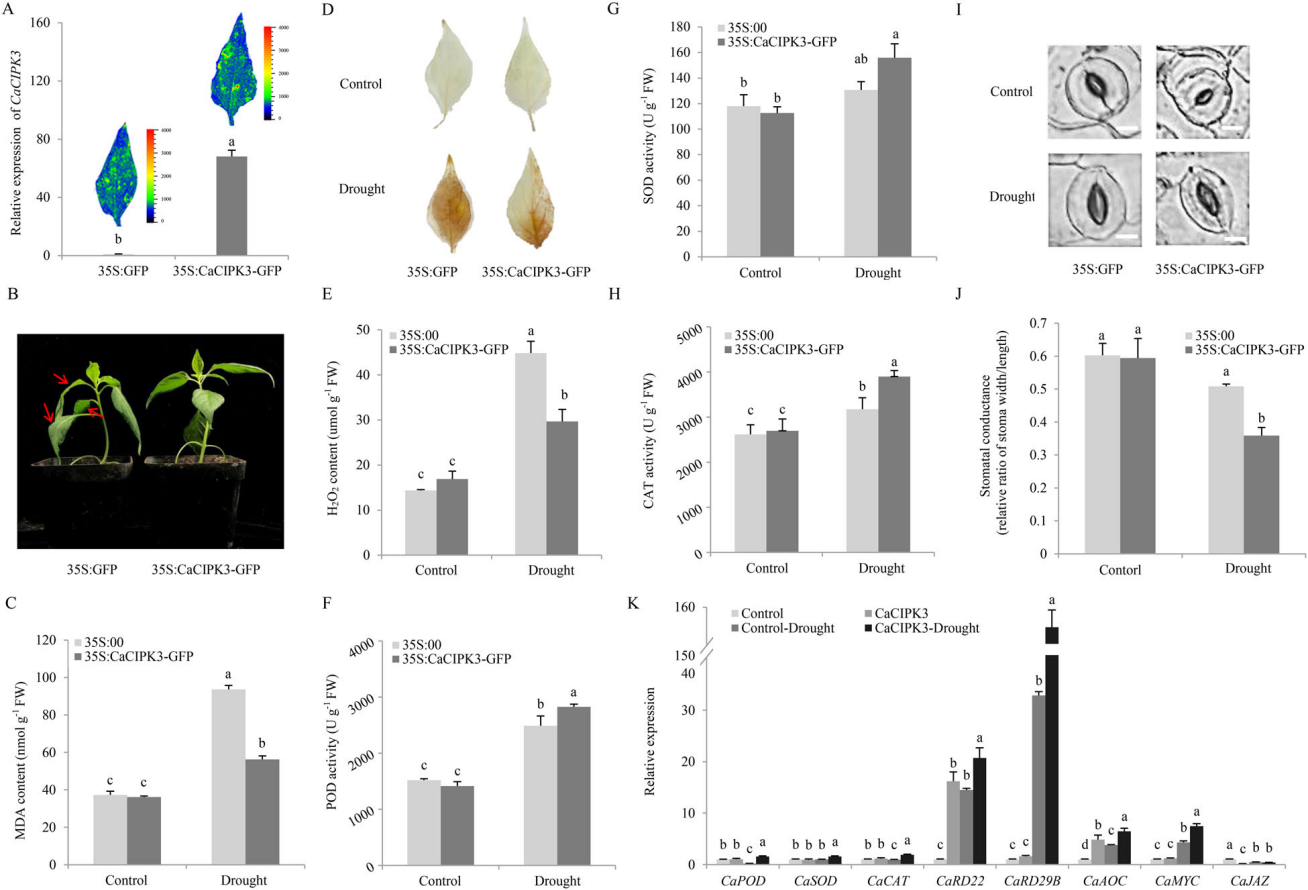
Transient overexpression of *CaCIPK*3 enhances drought resistance in pepper. **A** The expression of *CaCIPK3* and GFP signals in *CaCIPK3*-overexpressing and control plants. **B** Appearance of pepper after drought treatment. **C** MDA content. **D** H_2_O_2_ staining. **E** H_2_O_2_ content. **F** POD activity. **G** SOD activity. **H** CAT activity. **I**, **J** Stomatal aperture analysis. Scale bar, 10 µm. **K** Expression of *CaPOD*, *CaSOD*, *CaCAT*, *CaRD22*, *CaRD29B*, *CaAOC*, *CaMYC*, and *CaJAZ* in overexpression and control plants. All the values represent the means of three independent replicates ± SE (standard error). Means with different letters are significantly different according to Tukey’s test (*p* < 0.05)

The 35S:CaCIPK3-GFP vector was overexpressed in pepper leaves to investigate the effect of *CaCIPK3* in response to drought and MeJA. The 35S:GFP empty vector served as a control. The expression level of *CaCIPK3* was analyzed using qRT-PCR, and the GFP signals were visualized via Open FluorCam FC 800 at two days post inoculation (Fig. 3A). The plants were grown in dry soil at room temperature. Compared with the leaves transiently overexpressing *CaCIPK3-GFP*, the control leaves significantly wilted at 8 h (Fig. 3B). The leaves transiently overexpressing *CaCIPK3* had lower MDA and H_2_O_2_ contents but enhanced activities of the main ROS-scavenging enzymes compared with the control leaves (Fig. 3C–H). In addition, transient overexpression of *CaCIPK3* reduced stomatal opening in response to drought stress (Fig. 3I, J). We detected the expression of antioxidant-related genes, stress-related genes, and JA-related genes. The transcript levels of *CaRD22* and *CaAOC* increased, whereas that of *CaJAZ* was reduced in the leaves transiently overexpressing *CaCIPK3* under normal conditions (Fig. 3M). After drought stress, the expression of these genes, except *CaJAZ*, was significantly increased in *CaCIPK3*-overexpressing leaves.

The function of *CaCIPK3* in response to MeJA was also examined in leaves transiently overexpressing *CaCIPK3*. Leaf disks of the control leaves exhibited yellowing due to chlorophyll degradation, while those transiently overexpressing *CaCIPK3* remained green after MeJA treatment (Supplementary Fig. S2B). Collectively, these results demonstrate that *CaCIPK3* knockdown reduces the resistance of pepper to drought and MeJA. In contrast, *CaCIPK3* overexpression improves the tolerance of pepper to these stresses, indicating that *CaCIPK3* influences pepper resistance against drought and MeJA.

### *CaCIPK3* improves drought and MeJA tolerance in transgenic tomato

**Fig. 4:**
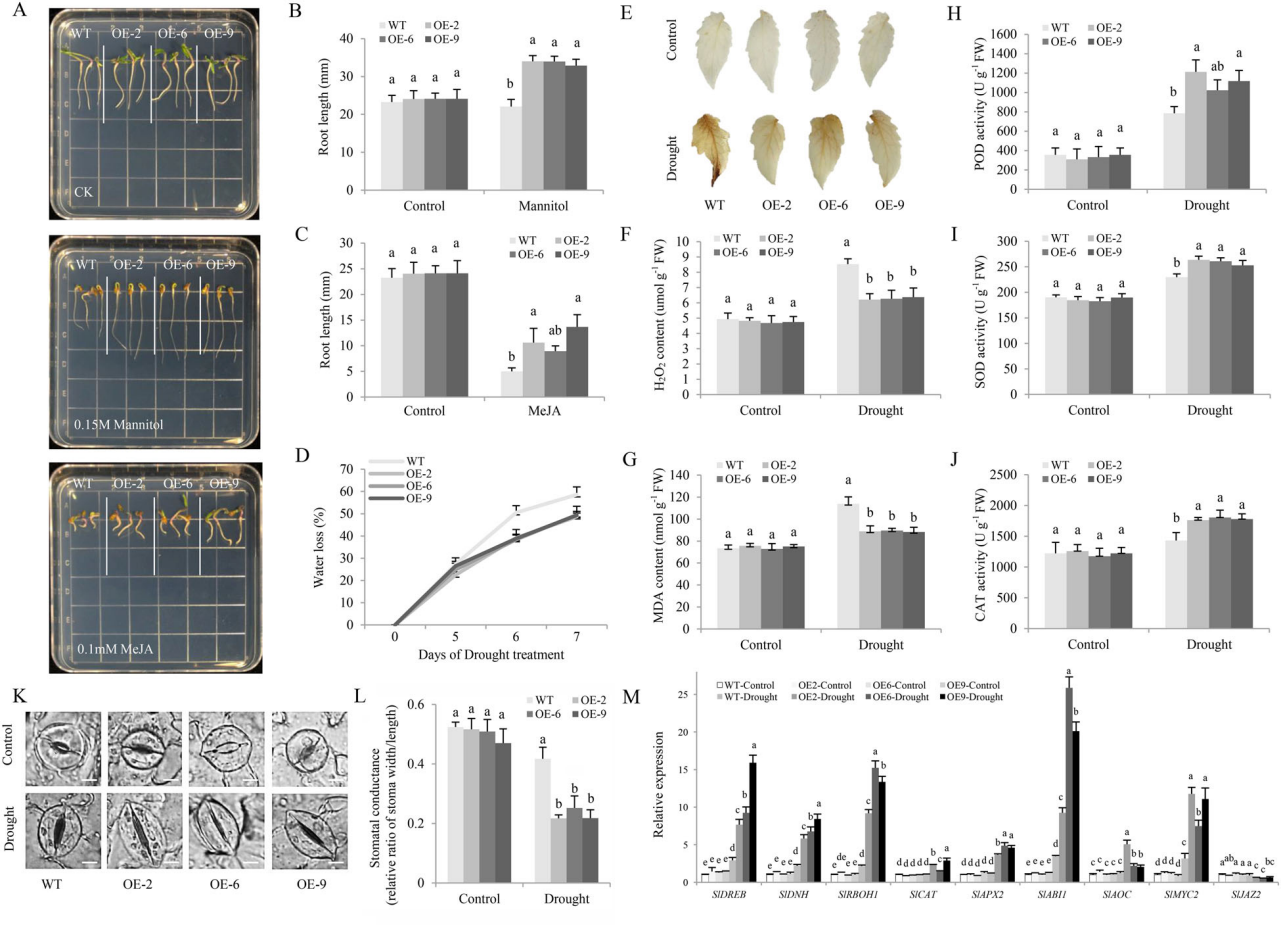
Overexpression of *CaCIPK3* improves drought tolerance in tomato. **A** Germinated seeds of WT and *CaCIPK3*-OE plants sown on MS medium containing 0.15 M mannitol and 0.1 mM MeJA. **B**, **C** Root length of WT and *CaCIPK3*-OE plants exposed to mannitol and MeJA. **D** Water loss. **E** H_2_O_2_ staining. **F** H_2_O_2_ content. **G** MDA content. **H** POD activity. **I** SOD activity. **J** CAT activity. **K**, **L** Stomatal aperture analysis. Scale bar, 5 µm. **M** Expression of *SlDREB*, *SlDHN*, *SlRBOH1*, *SlCAT*, *SlAPX2*, *SlABI1*, *SlAOC*, *SlMYC2*, and *SlJAZ2*. Data are the means ± SE (standard error) of three independent replicates. The letters (**a**–**e**) indicate significant differences based on Tukey’s test (*p* < 0.05)

To further elucidate the function of *CaCIPK3* in drought tolerance*, CaCIPK3* was heterologously expressed in tomato plants. Germinated seeds of homozygous lines (OE-2, OE-6, and OE-9 overexpressing *CaCIPK3*) and the WT line were sown on MS medium supplemented with 0.15 M mannitol or 0.1 mM MeJA and cultured for seven days (Supplementary Fig. S3A, Fig. 4A). There was a significant increase in root length in mannitol-exposed *CaCIPK3* transgenic seedlings compared to that of WT plants (Fig. 4B). The roots of the seedlings grew slowly under MeJA treatment, especially in the WT plants (Fig. 4C). Furthermore, water was withheld from four-week-old WT and OE plants for seven days to induce drought stress, while the control group was watered normally. After seven days, most leaves of the WT plants withered significantly, while those of the transgenic plants exhibited mild curling (Supplementary Fig. S3B). During drought stress, the WT plants showed a higher water loss rate than OE plants at the 6- and 7-day time points (Fig. 4D). Additionally, the levels of H_2_O_2_ and MDA were remarkably higher in the WT leaves than in the OE leaves (Fig. 4E–G). Although the contents of POD, SOD, and CAT increased in all the treatment groups, they were significantly higher in the transgenic plants (Fig. 4H–J). Moreover, the transgenic plants showed reduced leaf stomatal apertures compared with those of the WT plants (Fig. 4K, L). After drought stress, the genes related to stress, antioxidants, and JA were differentially expressed in the WT and OE plants (Fig. 4M). The expression patterns were consistent with those observed in pepper.

Leaf disks of the WT and OE plants were immersed in sterile water with or without 400 µM MeJA and incubated at room temperature for seven days (Supplementary Fig. S3C). The chlorophyll levels of WT leaves were lower than those of OE plants (Supplementary Fig. S3D). Corresponding to the phenotype, the transcription of chlorophyll and leaf senescence-related genes (*SlPAO* and *SlSGR*) was remarkably upregulated in the WT plants (Supplementary Fig. S3E). Together, these results further indicate that *CaCIPK3* overexpression improves tolerance to drought and MeJA. *CaCIPK3* potentially regulates drought tolerance through the antioxidant-dependent pathway and MeJA signaling.

### CaCIPK3 interacts with CaCBL2

**Fig. 5:**
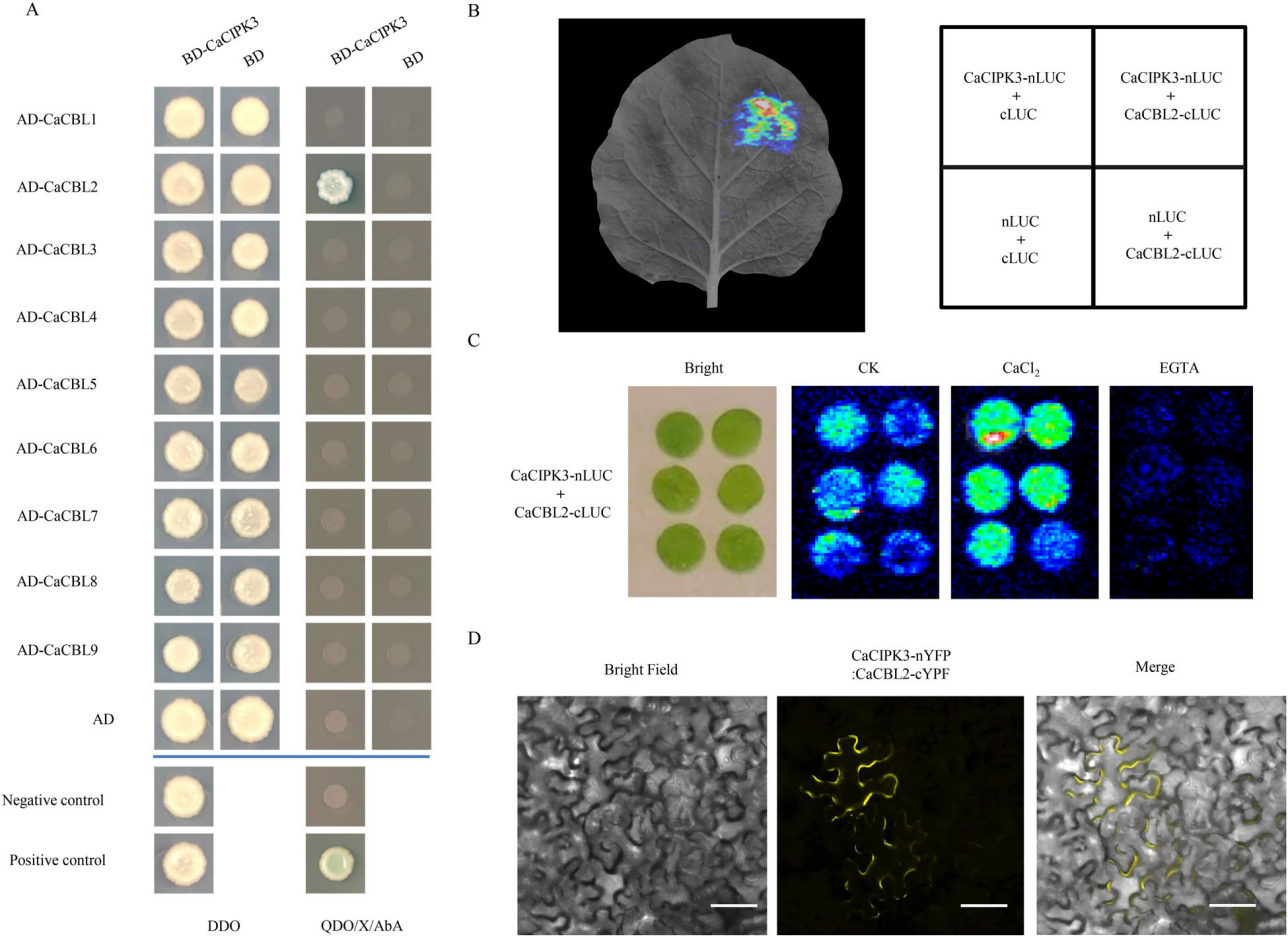
The interaction between CaCIPK3 and CaCBL2. **A** Y2H assay of CaCIPK3 and CaCBLs. Different protein combinations were tested on the screening medium (DDO and QDO with 20 µg/mL X-α-Gal and 125 ng/mL AbA). **B** LUC assay of CaCIPK3 and CaCBL2. **C** Effects of CaCl_2_ and EGTA treatment on the interaction between CaCIPK3 and CaCBL2. **D** BiFC analysis of CaCIPK3 and CaCBL2. Scale bar = 50 µm

Considering that CBLs activate CIPK enzyme activity, we examined the interactions between the CaCIPK3 protein and the nine pepper CBLs using yeast two-hybrid (Y2H) assays. Only CaCIPK3 and CaCBL2 grew on the QDOs with X-α-Gal and AbA screening medium (Fig. 5A). The interaction between CaCIPK3 and CaCBL2 was further examined using luciferase complementation imaging (LCI), which revealed that CaCIPK3 interacted with CaCBL2 (Fig. 5B). To determine whether the interaction of CaCIPK3 and CaCBL2 was based on Ca^2+^, we performed an LCI assay with CaCl_2_ and EGTA (chelated with Ca^2+^). The luminescence signal in the CaCl_2_ treatment was stronger than that in the control, whereas the luminescence signal in the EGTA treatment was almost undetectable, suggesting that the CaCBL2–CaCIPK3 interaction is facilitated by Ca^2+^ (Fig. 5C). Furthermore, bimolecular fluorescence complementation (BiFC) analysis showed that the yellow fluorescence signals were distributed on the plasma membrane (Fig. 5D).

### Knockdown of *CaCBL2* reduces drought resistance in pepper

Given that CaCIPK3 interacts with CaCBL2, we hypothesized that CaCIPK3 might increase drought tolerance in a CaCBL2-dependent signal-transduction pathway. A specific 272 bp sequence of *CaCBL2* was selected to successfully silence *CaCBL2* using a VIGS assay (Supplementary Fig. S4A). In comparison with the control plants, *CaCBL2*-silenced plants were more sensitive to mannitol stress (Supplementary Fig. S4B). The contents of MDA and H_2_O_2_ increased rapidly in the *CaCBL2*-silenced plants (Supplementary Fig. S4C–E). Simultaneously, POD, SOD, and CAT activities were higher in the control plants (Supplementary Fig. S4F–H). Furthermore, the stomatal morphology in the *CaCBL2*-silenced plants and control plants was examined. The stomatal apertures were reduced in the control plants compared to those in *CaCBL2*-silenced plants (Supplementary Fig. S4I–J). In addition, the transcript levels of the genes related to antioxidant, stress, and JA signaling were significantly higher in the control plants than in *CaCBL2*-silenced plants (Supplementary Fig. S4K). These findings indicate that *CaCBL2* weakens pepper resistance to drought by interacting with CaCIPK3.

### The *CaCIPK3* promoter is regulated by the CaWRKY1 and CaWRKY41 proteins

**Fig. 6:**
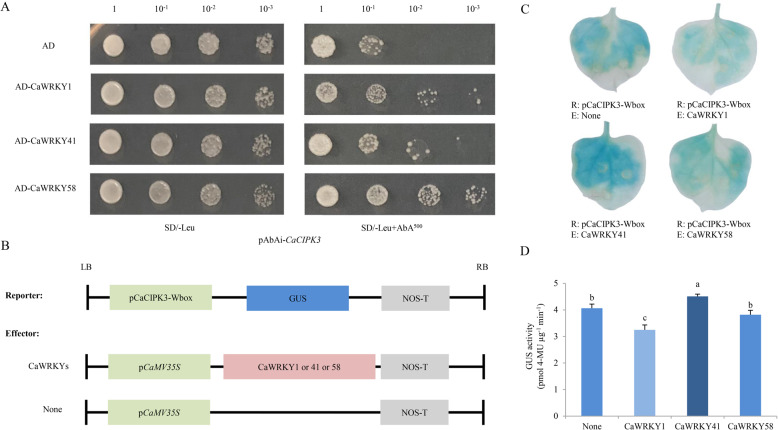
Analysis of *CaCIPK3* promoter activity after coinfection of tobacco leaves with CaWRKY1, CaWRKY41, and CaWRKY58. **A** Y1H assay demonstrating that CaWRKYs bind directly to the *CaCIPK3* promoter. pGADT7 served as a control. **B** Schematic representation of the reporter and effector. **C** Histochemical GUS staining in *N. tabacum* leaves. **D** GUS activity analysis. The data are the means of three independent replicates ± SE (standard error). Means with different letters are significantly different according to Tukey’s test (*p* < 0.05)

According to the W box in *CaCIPK3*_*pro*_, we speculated that WRKYs mediate activation of the *CaCIPK3* promoter. Three widely studied CaWRKYs (CaWRKY1, −41, −58) were selected to test their role in regulating the *CaCIPK3* promoter. A Y1H assay was used to detect whether CaWRKYs directly target the *CaCIPK3* promoter. The Y1H Gold yeast strain cotransformed with AD-CaWRKYs and pAbAi-*CaCIPK3* grew better than the empty control on medium containing 500 ng/mL AbA (Fig. 6A). These results indicate that the three CaWRKYs associate with the *CaCIPK3* promoter in vitro. To further examine the functions of CaWRKYs, the GUS transcript and enzymatic activity were analyzed in tobacco leaves. The reporter p*CaCIPK3*-Wbox-GUS combined with different effectors (35S:CaWRKYs) (Fig. 6B). The results suggest that CaWRKY1 suppresses the transcriptional activity of CaCIPK3, while CaWRKY41 induces CaCIPK3 activity (Fig. 6C, D). Notably, CaWRKY58 had no significant effect on CaCIPK3 activity. Collectively, these results suggest that CaWRKY1 and CaWRKY41 directly bind to the *CaCIPK3* promoter to regulate its activity.

## Discussion

Calcium is the core molecule of the signal transduction pathway and participates in regulating various environmental stimuli in plants. Drought stress accelerates the concentration of cytosolic Ca^2+^, thereby activating the special Ca^2+^ sensor CBL and its interacting protein kinases to promote downstream protein activity and gene transcription. The CBL–CIPK modules have been functionally characterized under biotic and abiotic stimuli, especially under salt stress, ion homeostasis (K^+^ and NO_3_^−^), and ABA treatment. However, the functions of pepper CIPKs in drought and MeJA stress remain unclear.

Herein, we characterized a multiple stress-induced protein kinase CaCIPK3. CaCIPK3 is homologous to AtCIPK14 and TaCIPK2 in *Arabidopsis* and wheat, respectively. AtCIPK14 phosphorylates ARABIDOPSIS TO´ XICOS EN LEVADURA 31 (ATL31) to mediate carbon and nitrogen nutrients^39^. Additionally, AtCIPK14 phosphorylates WHIRLY1 (WHY1) to regulate the subcellular localization and distribution of plastids and the nucleus, which influence chloroplast protein metabolism in leaf senescence^40,41^. TaCIPK2 interacts with TaCBL1, and its overexpression improves drought tolerance in plants^25^. In this study, the interaction between CaCBL2 and CaCIPK3 was verified using Y2H, BiFC, and LCI. CaCBL2 is closely related to *Arabidopsis* AtCBL1/-9, wheat TaCBL1, and rice OsCBL1. AtCBL1 and AtCBL9 interact with different CIPKs to regulate NO_3_^−^ homeostasis, K^+^ homeostasis, ABA signaling, and ROS signaling in the plasma membrane^14,16,17,42^. TaCBL1 interacts with TaCIPK23 and TaCIPK25 in the plasma membrane^43,44^. Plasma membrane-localized OsCBL1 modulates K^+^ and NO_3_^−^ signaling and influences root development^45,46^. Similarly, we verified that CaCBL2 recruits CaCIPK3 to the plasma membrane in a Ca^2+^-dependent manner.

In this study, RNA-seq and qRT-PCR analysis showed that *CaCIPK3* is regulated by abiotic stress and hormones. Salt, mannitol, and ABA induce the activity of *AtCIPK6* promoter in *Arabidopsis*. Overexpression of *AtCIPK6* improves salt resistance and influences ABA sensitivity^34^. Although some *cis*-acting elements related to ABA and MeJA were found in the *CaCIPK3* promoter, GUS activity was only enhanced by mannitol and MeJA in p*CaCIPK3*_*pro*_*:GUS*-overexpression lines. We speculated that *CaCIPK3* might play a vital function in response to drought and MeJA. The results of loss- and gain-of-function experiments showed opposite trends. Silencing of *CaCIPK3* enhanced the vulnerability of pepper to stresses and influenced several physiological and gene expression changes. In contrast, *CaCIPK3* overexpression enhanced drought resistance in pepper and tomato. Notably, *CaCBL2*-knockdown plants also exhibited decreased tolerance to drought stress. Drought induces excessive production of ROS in plants, which damages plant cellular structures and components^47^. ROS scavengers, including SOD, POD, and CAT, can convert excess and harmful ROS to harmless water in response to stress^48,49^. *TaCIPK2*-overexpressing lines showed greater ROS-scavenging abilities due to increased CAT and SOD activities in response to drought stress^25^. Drought stress causes stomatal closure facilitated by increased ABA production to relieve transpiration^50^. Similar to ABA, the phytohormone MeJA also induces stomatal closure^5^. In *Arabidopsis*, thioglucoside glucohydrolases TGG1 and TGG2 redundantly regulate guard cells via ROS production and Ca^2+^ elevation in ABA and MeJA signaling^51^. The grapevine CIPK member VaCIPK02 interacts with several CBLs and the ABA receptor PYL9. Overexpression of *VaCIPK02* improves drought tolerance by regulating ABA content and stomatal closure^28^. In this study, the activities of SOD, CAT, and POD increased, while H_2_O_2_ and MDA contents decreased in *CaCIPK3*-overexpression plants under drought stress. Concurrently, the stomatal apertures in the *CaCIPK3*-overexpression plants were reduced to prevent evaporation. The expression of stress-related genes, JA signaling genes, and antioxidant-related genes increased significantly in the *CaCIPK3*-overexpression plants under drought stress. In particular, transient overexpression of *CaCIPK3* induced the expression of *CaRD22* and *CaAOC* but restrained the transcript levels of *CaJAZ*, suggesting that *CaCIPK3* may facilitate plant drought tolerance by regulating the expression of these genes.

In this study, MeJA treatment significantly induced plant senescence and chlorophyll degradation. The biochemical pathway of chlorophyll degradation is regulated by chlorophyll catabolic genes (CCGs), including *PAO*, *NYE* (also called *SGR1*), and *PPH*. These genes coregulate chlorophyll degradation during leaf senescence^30,33,52^. The *nye1–1*, *pph-1*, and *pao1* mutant lines showed stay-green phenotypes in comparison with the wild type. AtMYC2/-3/-4 proteins belonging to the JA-signaling pathway enhance the transcriptional activity of *PAO*, *NYC1*, and *NYE1* by directly binding their promoters. These triple mutants of *mycs* showed the same phenotype as the *pao1* mutant^30^. In this study, *CaCIPK3*-overexpression plants exhibited stay-green phenotypes, while *CaCIPK3*-knockdown plants turned yellow under MeJA treatment. Consistent with the chlorophyll content, overexpression of *CaCIPK3* restrained the expression of CCGs to maintain a green phenotype.

**Fig. 7:**
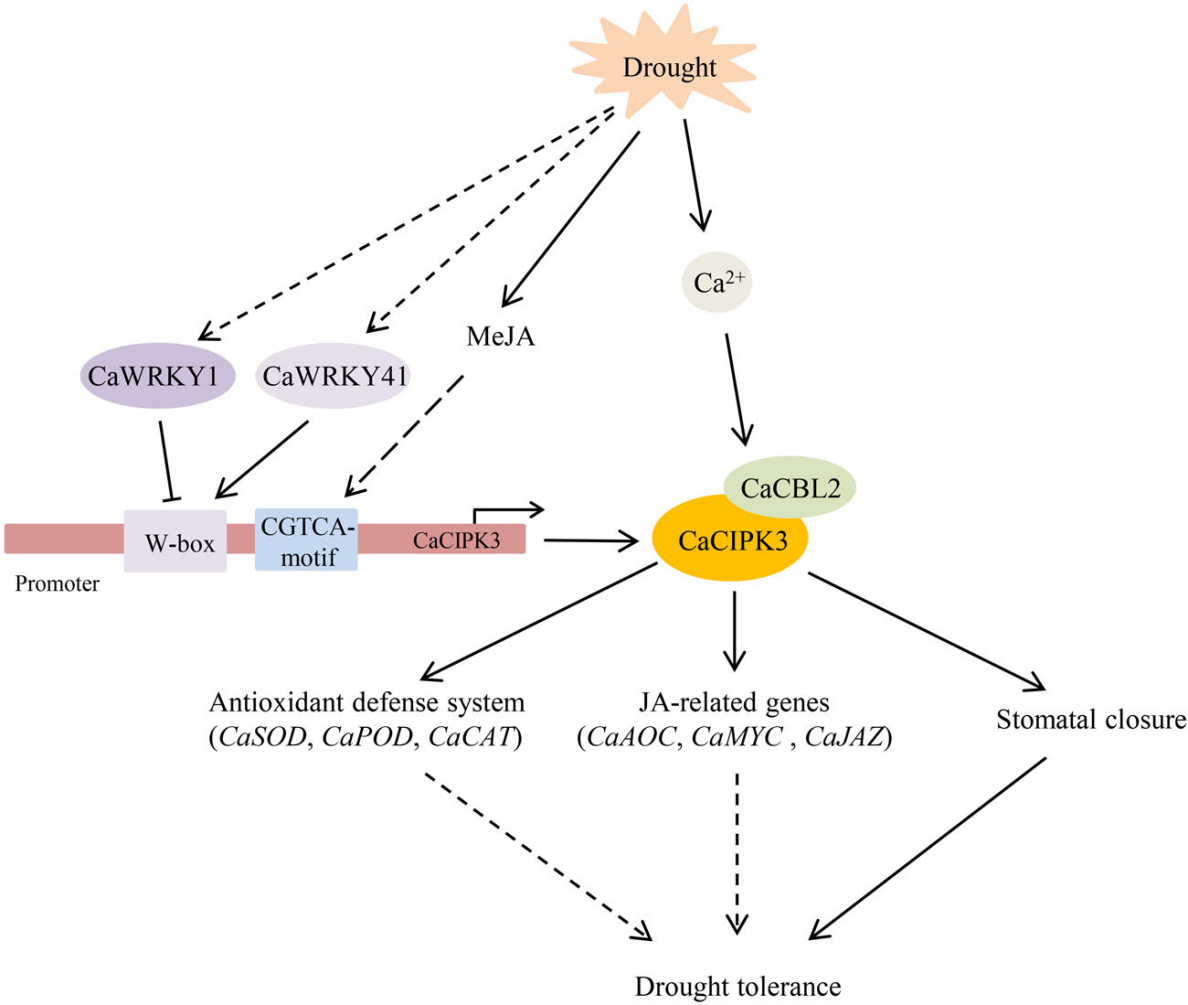
A schematic illustration of *CaCIPK3* expression under drought stress in pepper. Under drought stress, Ca^2+^ is rapidly induced and actives CaCBL2 to recruit CaCIPK3. CaWRKYs directly bind to the W-box in *CaCIPK3* promoter to regulate its expression. Overexpression of *CaCIPK3* enhances antioxidase activity, regulates the expression of JA-related genes and promotes stomatal closure, which contribute to the improvement of drought tolerance. Solid arrows indicate the direction of control and regulation, while dotted arrows indicate the possible mechanism

Many WRKY transcription factors have been identified to play significant roles in the response to drought stress in various plant species. *AtWRKY1* negatively regulates stomatal movement in response to drought stress^53^. *TaWRKY33* transgenic lines showed enhanced tolerance to drought^54^. Overexpression of *CaWRKY1* improved drought resistance in potato^55^. *WRKYs* also play essential roles in leaf senescence^56^. AtWRKY53 interacts with the JA-inducible protein ESR to adjust leaf senescence by JA and salicylic acid (SA) equilibrium^32^. Considering that a W-box exists in the *CaCIPK3* promoter, we examined the relationship between WRKYs and the *CaCIPK3* promoter. The transcript level of *CaWRKY58* was downregulated by exogenously applied MeJA^57^. *CaWRKY41* is involved in H_2_O_2_ accumulation in pepper^58^. Thus, CaWRKY1, CaWRKY41, and CaWRKY58 proteins were selected to verify their interactions with the promoter region of *CaCIPK3*. The results suggest that CaWRKY1 and CaWRKY41 regulate the activity of *CaCIPK3*. Few studies have focused on elucidating the connection between WRKY and CIPK. In wheat, TaWRKY9 binds to the *TaCIPK25* promoter and downregulates *TaCIPK25* expression^44^. In this study, CaCIPK3 interacted with CaCBL2 and was regulated by WRKYs, suggesting that *CaCIPK3*-mediated drought stress may involve positive or negative feedback regulation. In the working model (Fig. 7), when pepper suffers drought stress, the increase in cellular Ca^2+^ concentrations activates CaCBL2, which interacts with its partner CaCIPK3 and transduces the signal downstream. The expression levels of antioxidant-related genes and JA-related genes are influenced by *CaCBL2* and *CaCIPK3* under drought stress. However, the potential relationship of CaCBL2–CaCIPK3 and their downstream genes needs to be further studied. The activation of *CaCIPK3* is possibly regulated by CaWRKY1 and CaWRKY41. Additionally, MeJA signals bind to the CGTCA motif in the *CaCIPK3* promoter and induce stomatal closure during drought stress.

In conclusion, these findings indicate that *CaCIPK3* overexpression improves drought tolerance by regulating the antioxidant system and the expression of JA-related genes. Notably, *CaCIPK3*-regulated MeJA signaling potentially contributes to drought tolerance. These results provide physiological and molecular evidence to demonstrate the significance of *CaCIPK3* in plant drought tolerance.

## Materials and methods

### Plant materials and treatments

Pepper (*Capsicum annuum* L.) line P70, tomato (*Solanum lycopersicum* L.) cultivar Micro-Tom, and transgenic seedlings were cultivated in growth chambers under a 16/8-h day/night photoperiod and 25/22 °C day/night temperature. The peppers were treated with different stresses (0.1 M NaCl, 0.3 M mannitol, 0.1 mM MeJA, and 0.57 μM ABA) at the 6–8 true leaf stage in growth chambers as previously described^36^. Plants of uniform size were selected and subjected to different treatments.

### Gene expression analysis

Total RNA was extracted from different tissues of the experimental plants using an RNA extraction kit (Tiangen Biotech, Xi’an, China) according to the manufacturer’s instructions. RNA (1 µg per sample) was reverse transcribed to cDNA using a HiScript III 1st Strand cDNA Synthesis Kit (+gDNA wiper) (Vazyme Biotech Co., Ltd.). qRT-PCR was performed as previously described^36^. The reactions were performed in biological triplicates. Pepper *CaUBI3* and tomato *SlACTIN* genes were used as the internal controls for normalization of gene expression. Relative gene expression was calculated using the 2^−ΔΔCT^ method. The primers used are shown in Supplementary Table S2.

### Isolation and analysis of *CaCIPK3* and its promoter

The coding regions of *CaCIPK3* were cloned from the cDNA template of P70 seedlings. Multiple sequence alignment (MSA) analysis and phylogenetic tree construction were performed as previously reported^36^. Homologous proteins were acquired from NCBI (www.ncbi.nlm.nih.gov).

The *CaCIPK3* promoter was detected using PlantCARE (http://bioinformatics.psb.ugent.be/webtools/plantcare/html/)^59^. We cloned 1500 bp of the promoter from cDNA and inserted it into the pCAMBIA1381 vector. The pCAMBIA-*CIPK3*_*pro*_*:GUS* plasmid was introduced into *Agrobacterium tumefaciens* strain GV3101 using the freeze–thaw method^60^. The *Agrobacterium* culture was then used to transform wild-type *Arabidopsis thaliana* (Columbia-0) as described previously^61^. Transgenic *Arabidopsis* seeds were collected and screened on Murashige and Skoog (MS) medium containing 50 mg/L hygromycin. The T3 generations were used for subsequent experiments. A histochemical GUS assay was conducted to detect GUS activity as previously described^62^. Photographs showing GUS activity were taken using a microscope (SZX16, Olympus). GUS activities were quantified by ImageJ (National Institutes of Health) software.

### Subcellular localization of CaCIPK3

The cDNA sequences of *CaCIPK3* without stop codons were cloned from P70 leaves and inserted into the pVBG2307 vector harboring the green fluorescent protein (GFP) reporter gene. The fusion construct was introduced into *Agrobacterium* strain GV3101 containing the p19-silencing plasmid and coinfiltrated into tobacco (*Nicotiana tabacum*) leaves. GFP fluorescence was visualized using a microscope (BX63, Olympus).

### Virus-induced gene silencing (VIGS)

Several fragments of the *CaCIPK3* and *CaCBL2* genes were amplified by PCR using specific primers acquired from the Sol Genomics Network (http://vigs.solgenomics.net/). The unique fragments were confirmed using BLAST analysis and then inserted into the pTRV2 vector as previously described^36^. Approximately four weeks later, the expression of target genes was determined in pTRV2:CaCIPK3/CaCBL2 and pTRV2 plants using qRT-PCR. Silencing and control plants were used to conduct mannitol (300 mM) and MeJA (400 µM) stress assays.

### Overexpression of *CaCIPK3* in pepper and tomato plants

*A. tumefaciens* GV3101 cultures containing 35S:CaCIPK3-GFP or 35S:GFP plasmid vectors were infiltrated into the leaves of pepper plants to transiently overexpress *CaCIPK3* as previously reported^63,64^. GFP signals were captured using Open FluorCam (FC800, Photon System Instruments)^65^. Transgenic tomato (Micro-Tom) lines were generated using *Agrobacterium*-mediated transformation as described previously^66^. The putatively transformed tomato plants were screened on MS medium supplemented with 100 mg/L kanamycin. The lines that survived kanamycin selection were further screened using PCR to confirm the presence of the transgene. Seeds from wild-type (WT) plants and T3 generations of *CaCIPK3* were used to conduct drought and MeJA assays.

### Measurement of H_2_O_2_ and antioxidant enzyme activity

The content and activities of H_2_O_2_, superoxide dismutase (SOD), peroxidase (POD), and catalase (CAT) were determined at different wavelengths using detection kits (Solarbio, China) according to the manufacturer’s protocols. The production of H_2_O_2_ was detected using 3,3′-diaminobenzidine (DAB) staining.

### Physiological measurements and observations

The malondialdehyde (MDA) content was detected by thiobarbituric acid (TBA) using a modified protocol described previously^67^. The leaf chlorophyll (Chl) content was measured and calculated as described previously^68^. The water loss rate was calculated using the following formula: (FW–DW)/FW × 100% (FW: fresh weight; DW: dry weight).

Stomatal morphology was observed using a microscope (BX63, Olympus). Images were analyzed using ImageJ (National Institutes of Health) software.

### Protein-interaction assays

For the Y2H assay, a Matchmaker^TM^ Two-Hybrid System (Clontech, USA) was selected to identify the potential interacting proteins of CaCIPK3. The CDS of *CaCIPK3* was cloned into pGBKT7, whereas nine CaCBLs were cloned into pGADT7. The Y2HGold yeast strain containing BD-CaCIPK3 and AD-CaCBLs was screened on SD/-Leu/-Trp (DDO) medium. The positive clones were dotted on SD/-Leu/-Trp/-His/-Ade (QDO) medium supplemented with aureobasidin A (AbA) and X-α-Gal for selection and compared with the positive and negative controls. For the BiFC assay, *CaCIPK3*-pSPYNE, *CaCBL2*-pSPYCE, and p19 plasmids were infiltrated into *Nicotiana tabacum* as described by Xiao et al.^69^. Fluorescence was examined using a fluorescence microscope (BX63, Olympus) after 48 h of incubation. For the LCI assay, the CDSs of *CaCIPK3* and *CaCBL2* were cloned into pCAMBIA-nLUC and pCAMBIA-cLUC. The constructs were then introduced into *A. tumefaciens* GV3101 and coinfiltrated into the leaves of *N. tabacum*. After 48 h of incubation, luciferase activity was detected using a plant-imaging system (Lumazone Pylon 2048B, Princeton, USA) with an 8-min exposure. CaCl_2_ and EGTA treatments were performed as described in a previous study^70^.

### Y1H assay

A Y1H assay was performed to identify the function of the W-box element. The assay was conducted using the Matchmaker^TM^ One-Hybrid System (Clontech, USA) according to the manufacturer’s protocol. The unique sequence of the *CaCIPK3* promoter containing the W-box elements was inserted into the pAbAi vector as bait. Three *CaWRKY* genes (*CaWRKY1*, -*41*, and -*58*) were ligated into pGADT7 as prey.

### Evaluation of GUS activity

A unique sequence of the *CaCIPK3* promoter containing the W-box elements was inserted into the pCAMBIA1381-GUS vector as the reporter plasmid (pCaCIPK3-Wbox-GUS). Three *CaWRKY* genes (*CaWRKY1*, -*41*, and -*58*) were cloned into the vector under the control of the CaMV35S promoter as effector plasmids. *A. tumefaciens* GV3101 harboring the reporter and effector plasmids was infiltrated into the leaves of *N. tabacum*. Histochemical GUS assays were performed as previously described^62^. Fluorescence was measured at 365 nm for excitation and 455 nm for emission using a Tecan Infinite M200 Pro Reader (Tecan, Switzerland). The specific GUS activity is expressed in pmol 4-MU µg^−1^ of protein min^−1^.

### Statistical analysis

Data analysis was performed using SPSS 22.0 software. One-way analysis of variance (ANOVA) was used to analyze the differences between various treatments. Significant differences were determined at *p* < 0.05 according to Tukey’s test. All data are presented as the means ± SE (standard error).

## Supplementary information

Supplementary information

Supplementary Fig. S1

Supplementary Fig. S2

Supplementary Fig. S3

Supplementary Fig. S4

## Data Availability

The data that support the results are provided in this paper and its supplementary files.
