# Supplementary material for: The CaCIPK3 gene positively regulates drought tolerance in pepper
Source: Hortic Res. 2021 Oct 1;8:216. doi: 10.1038/s41438-021-00651-7 (PMC8484583; doi:10.1038/s41438-021-00651-7)
Supplement: Supplementary file 1 — Supplementary information [file 41438_2021_651_MOESM1_ESM.doc]

**Supplementary information**

This file contains Supplementary Figures S1-S4 and Tables S1-S2.


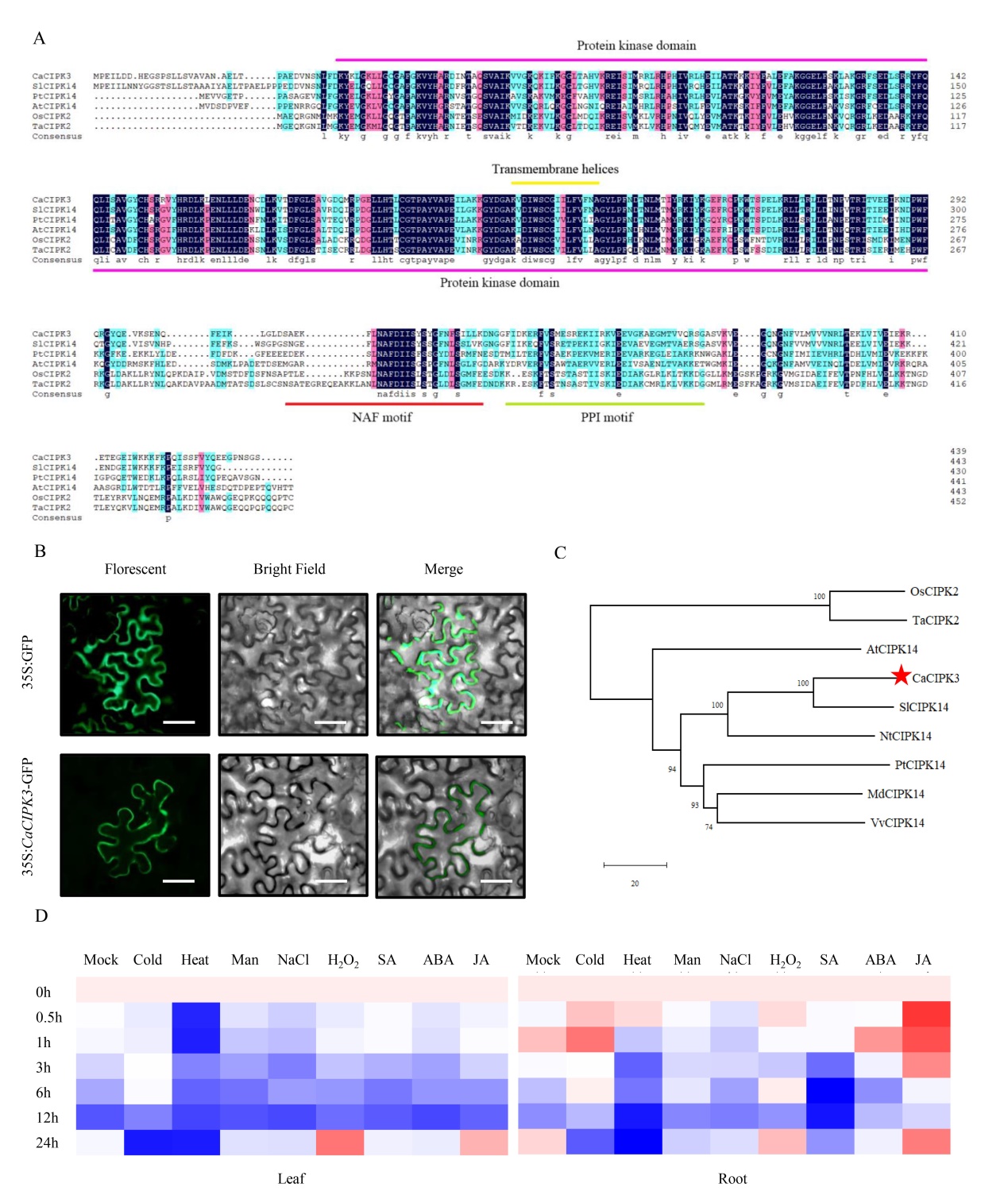


**Supplementary Fig. S1. Analysis of CaCIPK3.** (A) Multiple sequence alignment (MSA) analysis. (B) Subcellular localization of CaCIPK3 in *N. tabacum* epidermal cells. Scale bar, 50 µm. (C) The phylogenetic analysis. (D) Expression patterns of *CaCIPK3* in RNA-seq.


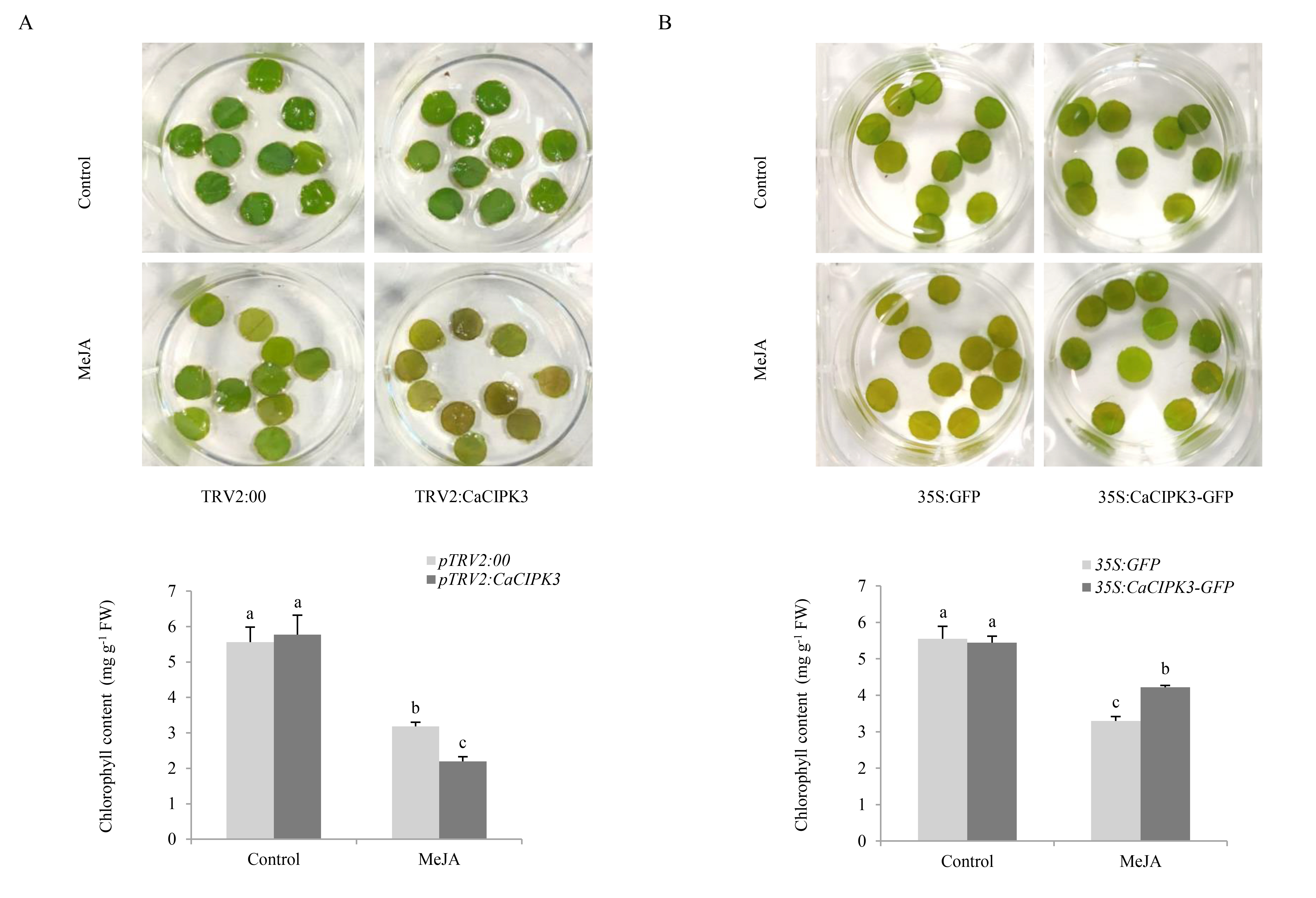


**Supplementary Fig. S2**. Appearance and chlorophyll content of *CaCIPK3-*silenced plants (A) and transient overexpression of *CaCIPK*3 (B) in pepper under 400µM MeJA condition. Data are means of three independent replicates ± SE (standard error). The letters show significant differences (Tukey’s test, p < 0.05).


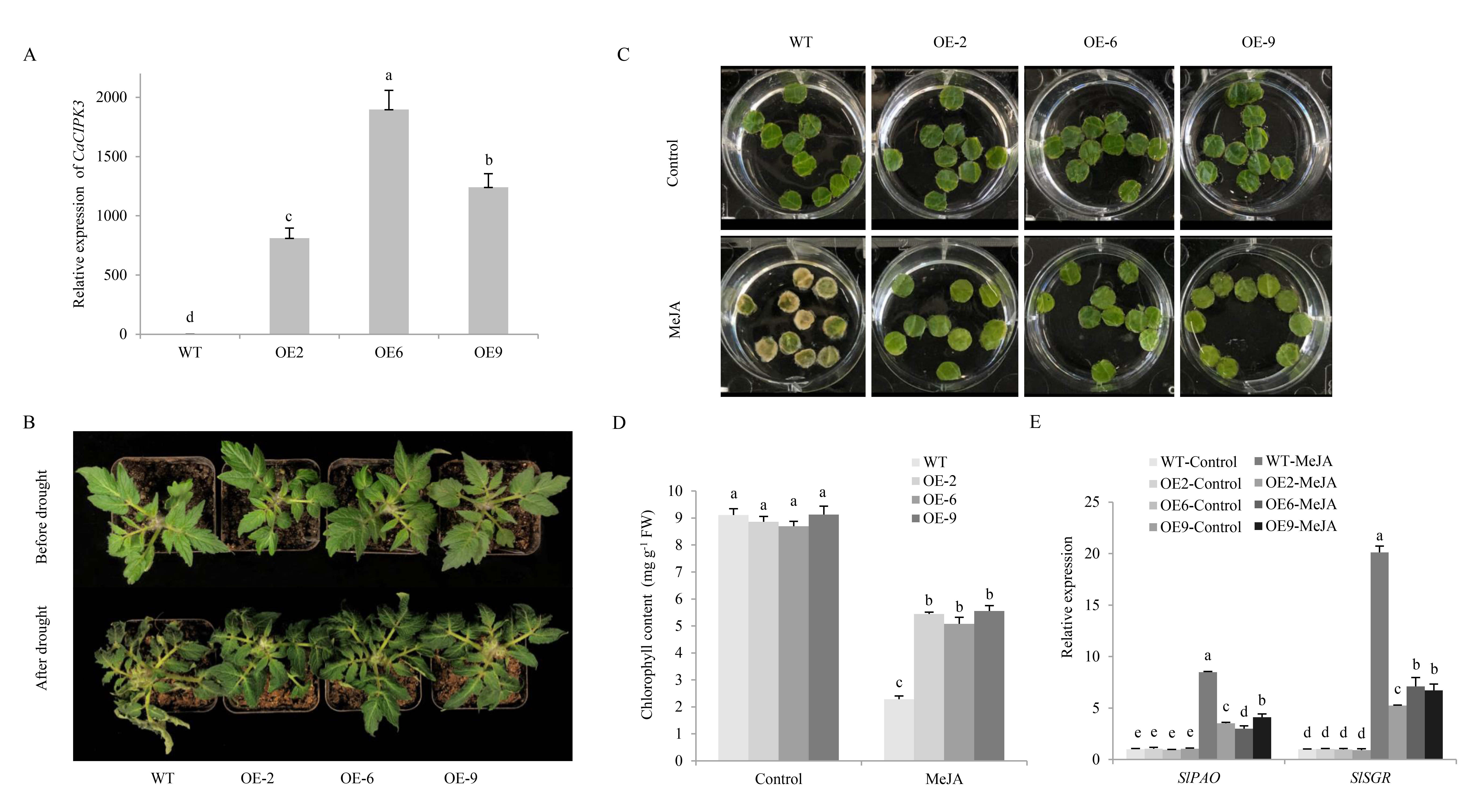


**Supplementary Fig. S3**. Overexpression of *CaCIPK3* enhances drought and MeJA tolerance in tomato. (A) The relative expression of *CaCIPK3* in tomato. (B) Appearance under drought stress. (C) Phenotype under MeJA treatment. (D) Chlorophyll content. (E) The expression levels of *SlPAO* and *SlSGR*. Values are means ± SE (standard error) of three independent replicates and significant differences were analzed by Tukey’s test (p < 0.05).


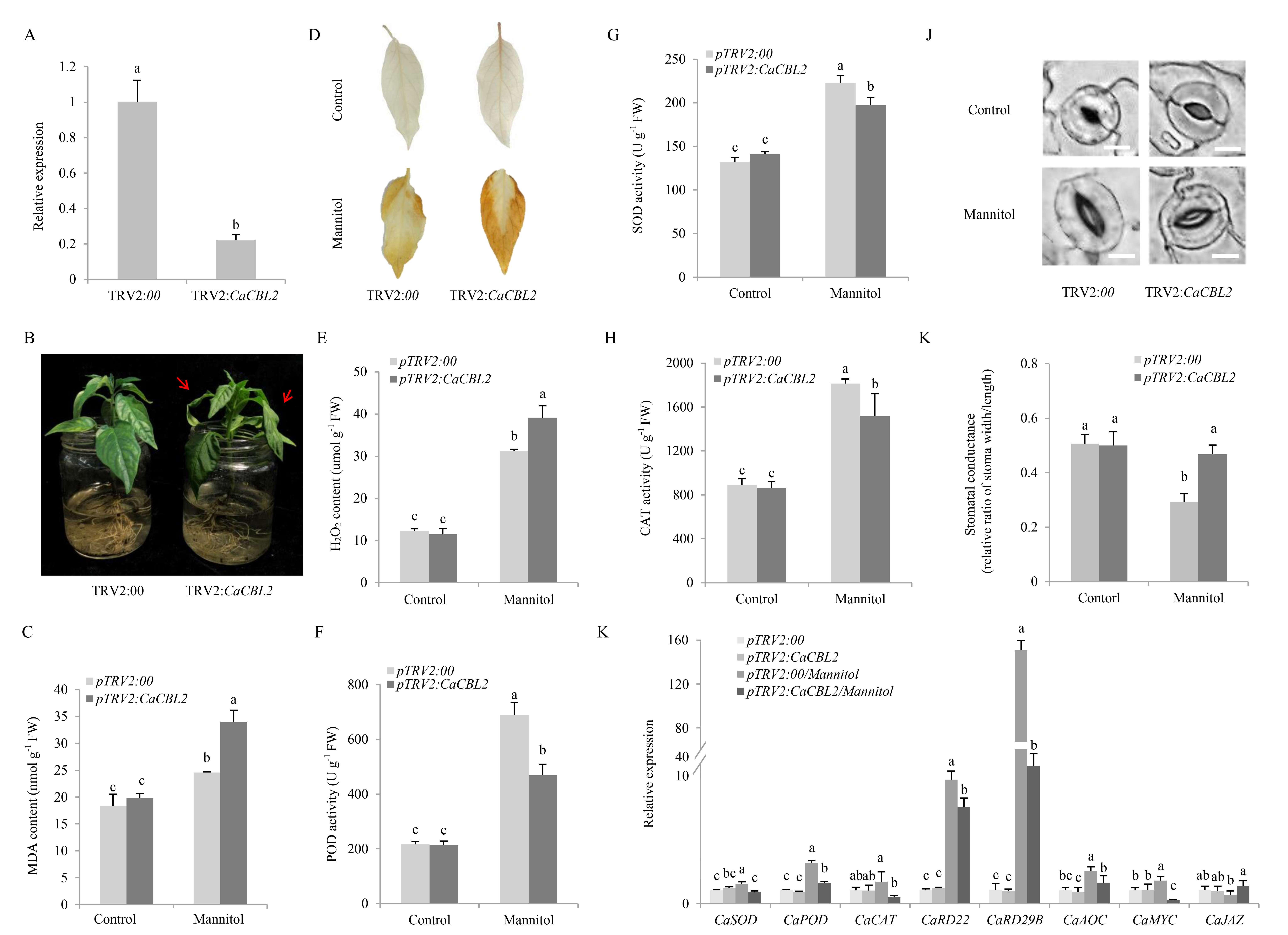


**Supplementary Fig. S4.** Silencing of *CaCBL2* reduces pepper’s tolerance to mannitol. (A) The relative expression of *CaCBL2* in silenced and control plants. (B) Appearance of pepper exposed to 300 mM mannitol. (C) MDA content. (D) H_2_O_2_ staining. (E) H_2_O_2_ content. (F) POD activity. (G) SOD activity. (H) CAT activity. (I, J) Stomatal aperture analysis. The scale bar represents 10 µm. (K) Expressions of *CaSOD*, *CaPOD*, *CaCAT*, *CaRD22*, *CaRD29B*, *CaAOC*, *CaMYC* and *CaJAZ* in silenced and mock plants under normal and mannitol conditions. All data are means of three independent replicates ± SE (standard error) and significant differences (Tukey’s test, p < 0.05).

| **Supplementary Table. S1.Putative *cis*-acting elements of the promoter of *CaCIPK3*** | | | |
| --- | --- | --- | --- |
| ***Cis*-acting element** | **Num.** | | **Function** |
| **Abiotic stress responsive** |  |  | |
| ABRE | 2 | *cis*-acting element involved in the abscisic acid responsiveness | |
| ARE | 2 | *cis*-acting regulatory element essential for the anaerobic induction | |
| CGTCA-motif | 4 | *cis*-acting regulatory element involved in the MeJA-responsiveness | |
| GC-motif | 1 | Enhancer-like element involved in anoxic specific inducibility | |
| WUN-motif | 1 | Wound-responsive element | |
| **Light responsive** |  |  | |
| AAAC-motif | 1 | light responsive element | |
| Box 4 | 1 | Part of a conserved DNA module involved in light responsiveness | |
| G-Box | 1 | *cis*-acting regulatory element involved in light responsiveness | |
| G-box | 1 | *cis*-acting regulatory element involved in light responsiveness | |
| GATA-motif | 1 | Part of a light responsive element | |
| I-box | 1 | Part of a light responsive element | |
| MRE | 1 | MYB binding site involved in light responsiveness | |
| Sp1 | 1 | light responsive element | |
| TCT-motif | 1 | Part of a light responsive element | |
| **Others** |  |  | |
| W-box | 1 | WRKY binding site | |
| CCAAT-box | 1 | MYBHv1 binding site | |
| MSA-like | 1 | *cis*-acting element involved in cell cycle regulation | |

| **Supplementary Table. S2. Primers used in this study** | |
| --- | --- |
| Gene | Sequence (5’ to 3’) |
| qPCR-CaCIPK3-F | CACCGTGATTTGAAACTGGAGA |
| qPCR-CaCIPK3-R | CCGCAAAGTGTATGGAGCAACT |
| p1381-CaCIPK3-F | CGGGATCCCAGATAACCAATTAAACTGTGA |
| p1381-CaCIPK3-R | AACTGCAGACAGACCAGTAAAACAGTAACC |
| 2307GFP-CaCIPK3-F | GCTCTAGAATGCCAGAGATCTTAGATGACC |
| 2307GFP-CaCIPK3-R | CGGGGTACCGCTACCAGAATTTGGTCCTTC |
| TRV2-CaCIPK3-F | GCTCTAGAGGCGAGTTGCTCCATACAC |
| TRV2-CaCIPK3-R | GGGGTACCTTGAAACCACGGGTCATTC |
| TRV2-CaCBL2-F | GCTCTAGAATGGGCTGTTTTAGCTCTAAG |
| TRV2-CaCBL2-R | GGGGTACCAAATCAATGACTCCTCTTTGCT |
| qPCR-CaCBL2-F | AAGCAAGGAAGAGTTCCAGTTAG |
| qPCR-CaCBL2-R | CTGAGCCATCCAGATCATACAG |
| BD-CaCIPK3-F | CCCATATGATGCCAGAGATCTTAGATGACC |
| BD-CaCIPK3-R | GCGTCGACTCAGCTACCAGAATTTGGTCC |
| AD-CaCBL1-F | CCCATATGATGGGCTGCTTTAATTCTAAA |
| AD-CaCBL1-R | CGGGATCCTTATGTAGCAACTTCATCAACTTC |
| AD-CaCBL2-F | CCCATATGATGGGCTGTTTTAGCTCTAAG |
| AD-CaCBL2-R | CGGGATCCTCATGTAGCTACTTCATCAACTTC |
| AD-CaCBL3-F | CCCATATGATGGGTTGTGCTTTAAGGAA |
| AD-CaCBL3-R | CGGGATCCTCATTCTAGTGTATTCACCACAAA |
| AD-CaCBL4-F | CCCATATGATGGGCTGCTTTCACTCA |
| AD-CaCBL4-R | CGGGATCCCTATCTGGTATTAGCATGAGCC |
| AD-CaCBL5-F | CCCATATGATGGATTCTACCCGCAGTT |
| AD-CaCBL5-R | CGGGATCCTCACAACAAATGGCTTTTCT |
| AD-CaCBL6-F | CCCATATGATGGGCTGTCTTCATTCTAC |
| AD-CaCBL6-R | CGGGATCCTTATAGTTGGGTAGCAACTTCA |
| AD-CaCBL7-F | CCCATATGATGCTGCAGTGCTTAGGTT |
| AD-CaCBL7-R | CGGGATCCTCAGGTGTCTTCAACTCTTGA |
| AD-CaCBL8-F | CCCATATGATGTCGCATTGCTTTGAG |
| AD-CaCBL8-R | CGGGATCCTCAGGTATCTGGAACTCTCG |
| AD-CaCBL9-F | CCCATATGATGCATGCACTAACGGGA |
| AD-CaCBL9-R | CGGGATCCTCAGTTTTCAAACACTAGTTGTGA |
| nLUC-CaCIPK3-F | GACGAGCTCGGTACCATGCCAGAGATCTTAGATGACC |
| nLUC-CaCIPK3-R | CGAGATCTGGTCGACGCTACCAGAATTTGGTCCTTC |
| cLUC-CaCBL2-F | TCCCGGGGCGGTACCATGGGCTGTTTTAGCTCTAAG |
| cLUC-CaCBL2-R | GCTCTGCAGGTCGACTCATGTAGCTACTTCATCAACTT |
| NE-CaCIPK3-F | CCCAGGCCTACTAGTGGATCCATGCCAGAGATCTTAGATGACC |
| NE-CaCIPK3-R | CCCGGGAGCGGTACCCTCGAGTCAGCTACCAGAATTTGGTCC |
| CE-CaCBL2-F | TGGCGCGCCACTAGTGGATCCATGGGCTGTTTTAGCTCTAAG |
| CE-CaCBL2-R | CCCGGGAGCGGTACCCTCGAGTGTAGCTACTTCATCAACTTCTGA |
| pAbAI-CaCIPK3-F | CCAAGCTTTTGACCTAAATAAGAGCATC |
| pAbAI-CaCIPK3-R | CCCTCGAGGTCAAATACCCACTTTATTCTATGTC |
| AD-CaWRKY1-F | GCCATGGAGGCCAGTGAATTCATGGAATTTACCAGTTTGGTTG |
| AD-CaWRKY1-R | ACGATTCATCTGCAGCTCGAGTTACCATCTGCCCGTCTGA |
| AD-CaWRKY41-F | GCCATGGAGGCCAGTGAATTCATGGAGAAAGTTAAAGGATTGG |
| AD-CaWRKY41-R | ACGATTCATCTGCAGCTCGAGTTAACTAAAGAACTCTTCAATGTCAA |
| AD-CaWRKY58-F | GCCATGGAGGCCAGTGAATTCATGGGAGAAACCGGGGGAGA |
| AD-CaWRKY58-R | ACGATTCATCTGCAGCTCGAGACGCCAAGTATCTTTGGCTG |
| p1381-WB-CIPK3-F | CGGAATTCTTGACCTAAATAAGAGCATC |
| p1381-WB-CIPK3-R | AACTGCAGGTCAAATACCCACTTTATTCTATGTC |
| 2307GFP-CaWRKY1-F | GCTCTAGAATGGAATTTACCAGTTTGGTTG |
| 2307GFP-CaWRKY1-R | GGGGTACCCCATCTGCCCGTCTGATTA |
| 2307GFP-CaWRKY41-F | GCTCTAGAATGGAGAAAGTTAAAGGATTGG |
| 2307GFP-CaWRKY41-R | GGGGTACCACTAAAGAACTCTTCAATGTCAAAT |
| 2307GFP-CaWRKY58-F | GCTCTAGAATGGGAGAAACCGGGGGAGA |
| 2307GFP-CaWRKY58 | GGGGTACCACGCCAAGTATCTTTGGCTG |
| qPCR-CaRD29B-F | ATGGAGGCACAACTGCACCGTC |
| qPCR-CaRD29B-R | GGCCCACCATGAACTTCTGCAC |
| qPCR-CaSOD-F | CTCTGCCATAGACACCAACTT |
| qPCR-CaSOD-R | CCAAGTTCGGTCCTTTAATAA |
| qPCR-CaPOD-F | GCAGCATTCCTCCTCCTACT |
| qPCR-CaPOD-R | ATTTCTTTGCCTTGTTGTTG |
| qPCR-CaCAT-F | TGTTGCTGGTGTTGGTGTTGGT |
| qPCR-CaCAT-R | GCCTCTCCTAGACGGCCTTTCA |
| qPCR-CaRD22-F | TGCTTTCTTGCCTCGTCA |
| qPCR-CaRD22-R | AACACCTGGTTCTTCACATTCT |
| qPCR-CaAOC-F | TACAGGGACCGTACTTAACCTA |
| qPCR-CaAOC-R | CTGCAATTTTACTTGACCGGAA |
| qPCR-CaJAZ-F | AGTAACAACGCCAACAATACAC |
| qPCR-CaJAZ-R | TCATTTGATGTTCCTGGATGGA |
| qPCR-CaMYC-F | GCTTCAGACGATTGTTTGTGTA |
| qPCR-CaMYC-R | CATTCAACGAATCCTTAGCAGC |
| qPCR-CaUBI3-F | TGTCCATCTGCTCTCTGTTG |
| qPCR-CaUBI3-R | CACCCCAAGCACAATAAGAC |
| qPCR-SlDHN-F | TGGTTTGTTTGATTTCATTG |
| qPCR-SlDHN-R | AGTTTCTTTTCCTCCTCCTT |
| qPCR-SlDREB-F | GACTCATTGCCTCGCCCA |
| qPCR-SlDREB-R | TTTCACCCAGTTCCTCCG |
| qPCR-SlABI1-F | GATGGGCTATGGGATGTCTT |
| qPCR-SlABI1-R | CTTGAGCAGCAGGATCTACG |
| qPCR-SlRBOH1-F | AAAGGAGTGGAGGGTGTGAC |
| qPCR-SlRBOH1-R | ACTTAATGAGGCTCCGCCTA |
| qPCR-SlAPX2-F | TCAGTGATCCTGCTTTCCGC |
| qPCR-SlAPX2-R | TGTCACCACCCTCCCAACTCT |
| qPCR-SlCAT-F | TCCTTGTCGTCCTGCTGAG |
| qPCR-SlCAT-R | TTGATGTATCTGTCTTGCCTGTC |
| qPCR-SlAOC-F | CAGCAGGACTCTGCATTCTG |
| qPCR-SlAOC-R | CGGTGACGGCTAGGTAAGTT |
| qPCR-SlMYC2-F | AATTTGGGGCTGGGGAGGAGAAT |
| qPCR-SlMYC2-R | CTCCACCTGACTTCATGCCCGAA |
| qPCR-SlJAZ2-F | GCAAGGTGACCGGACAGAAATCTC |
| qPCR-SlJAZ2-R | TGTGGTGGTAGTAGCTGTTTGTTGA |
| qPCR-SlPAO-F | GCATTCCGAAATTGGCTTAGAC |
| qPCR-SlPAO-R | GCTAATCCAGCACTTATAATTGC |
| qPCR-SlSGR-F | AACTCCCTGTGGTTCTCAAG |
| qPCR-SlSGR-R | GGAAAGCAACAGGCACAAGC |
| qPCR-SlACTIN-F | GGGATGGAGAAGTTTGGTGGTGG |
| qPCR-SlACTIN-R | CTTCGACCAAGGGATGGTGTAGC |
