# Supplementary figures and images for: The CaCIPK3 gene positively regulates drought tolerance in pepper
Source: Hortic Res. 2021 Oct 1;8:216. doi: 10.1038/s41438-021-00651-7 (PMC8484583; doi:10.1038/s41438-021-00651-7)

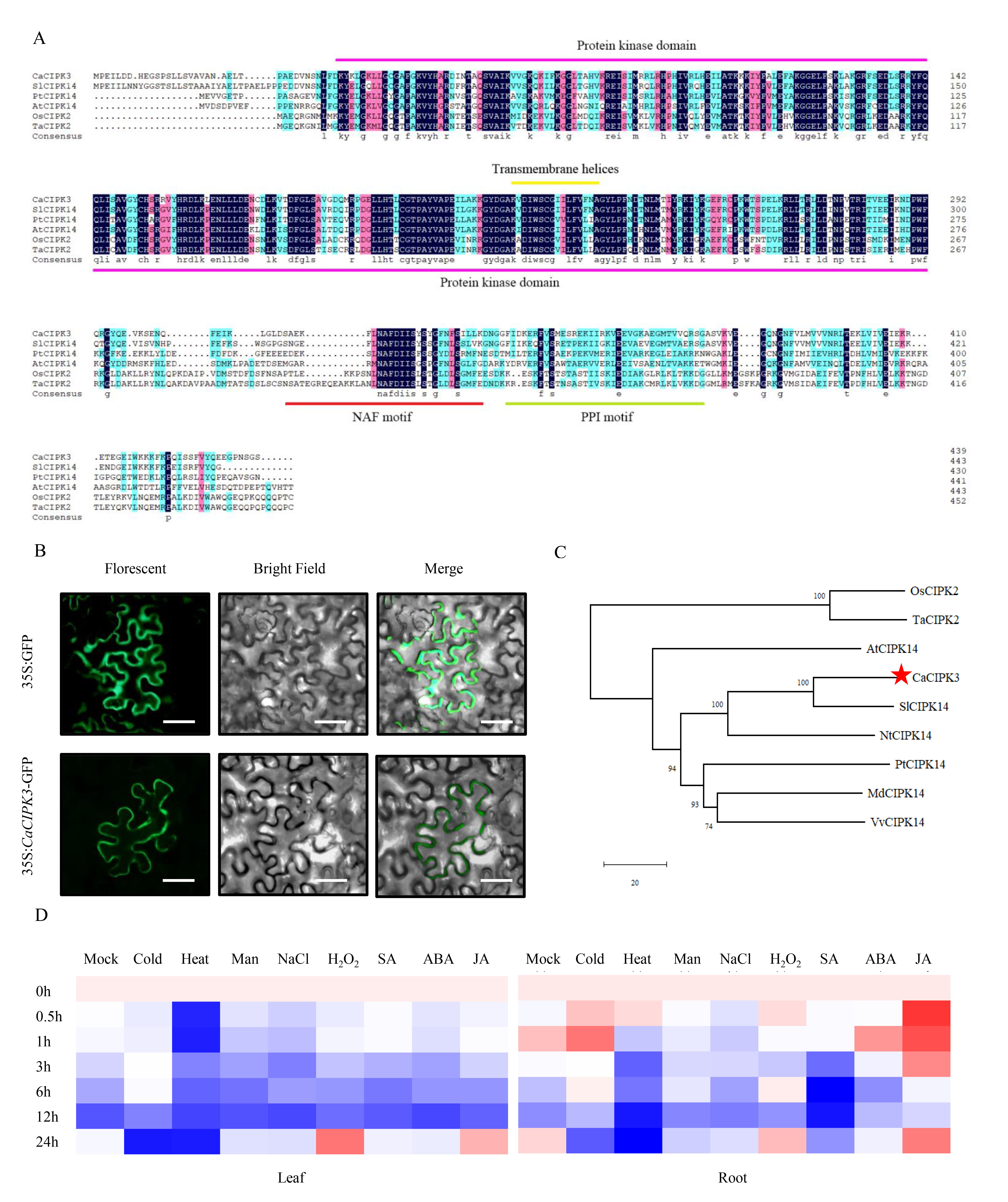

Supplement: Supplementary file 2 — Supplementary Fig. S1 [file 41438_2021_651_MOESM2_ESM.jpg]

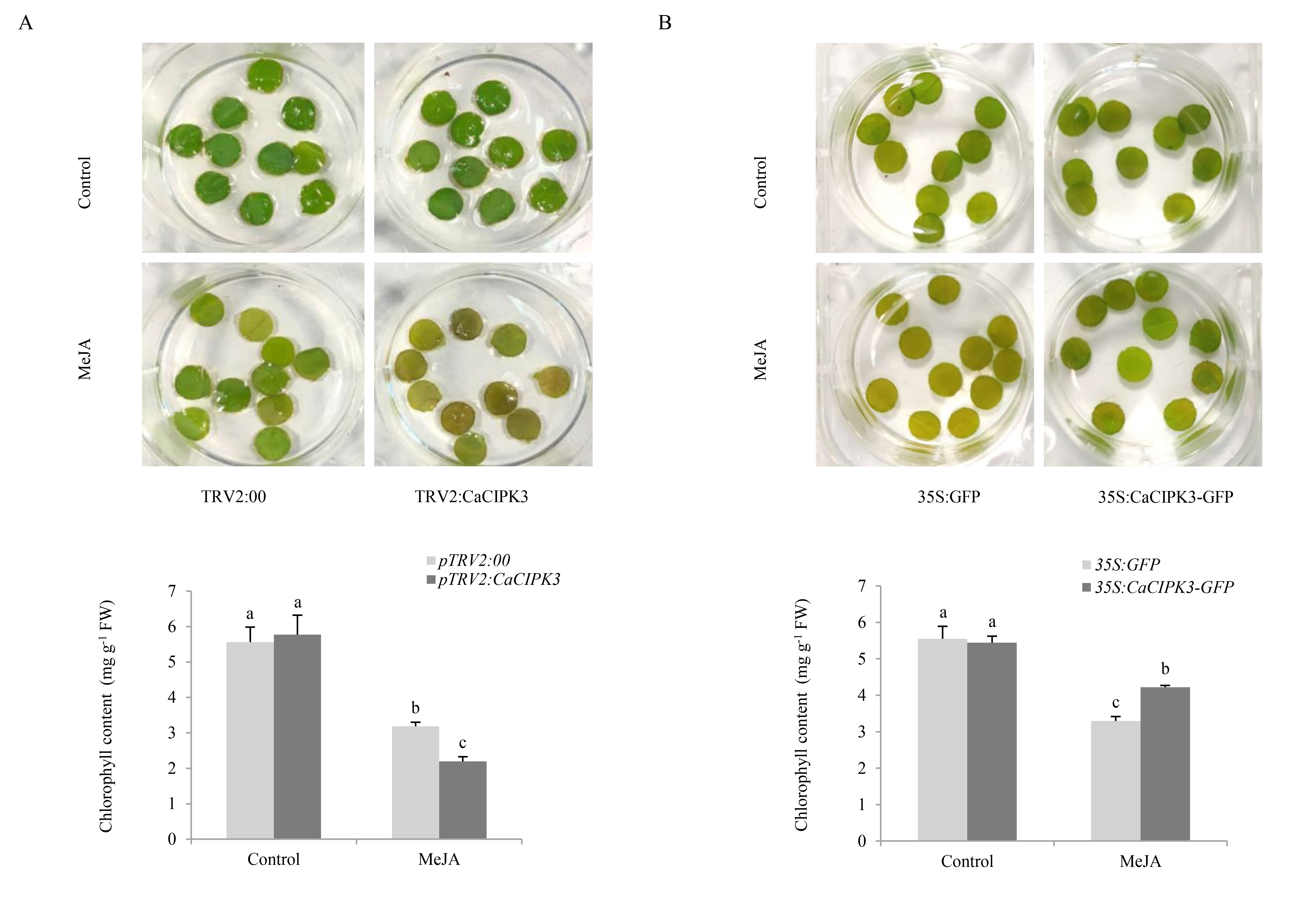

Supplement: Supplementary file 3 — Supplementary Fig. S2 [file 41438_2021_651_MOESM3_ESM.jpg]

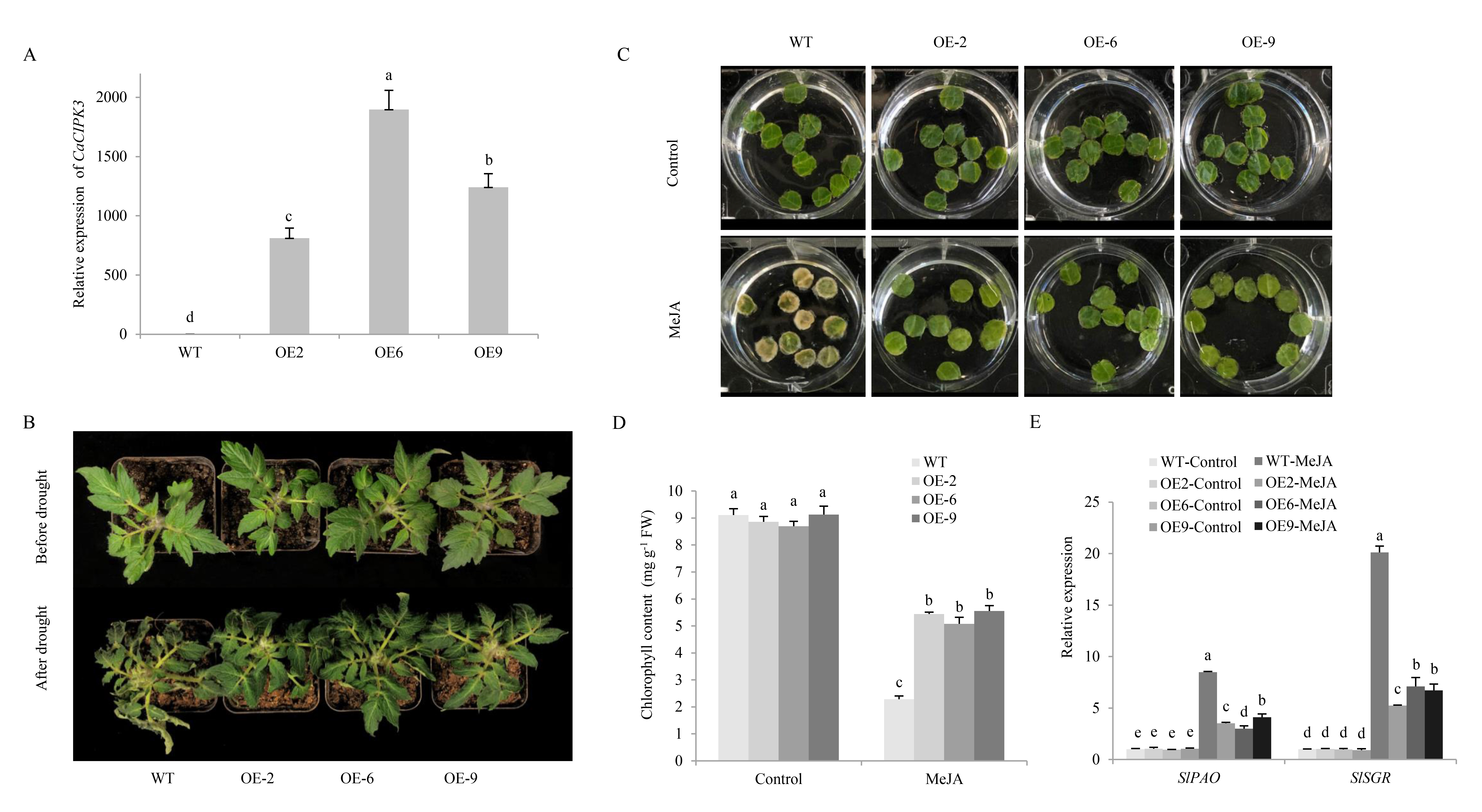

Supplement: Supplementary file 4 — Supplementary Fig. S3 [file 41438_2021_651_MOESM4_ESM.jpg]

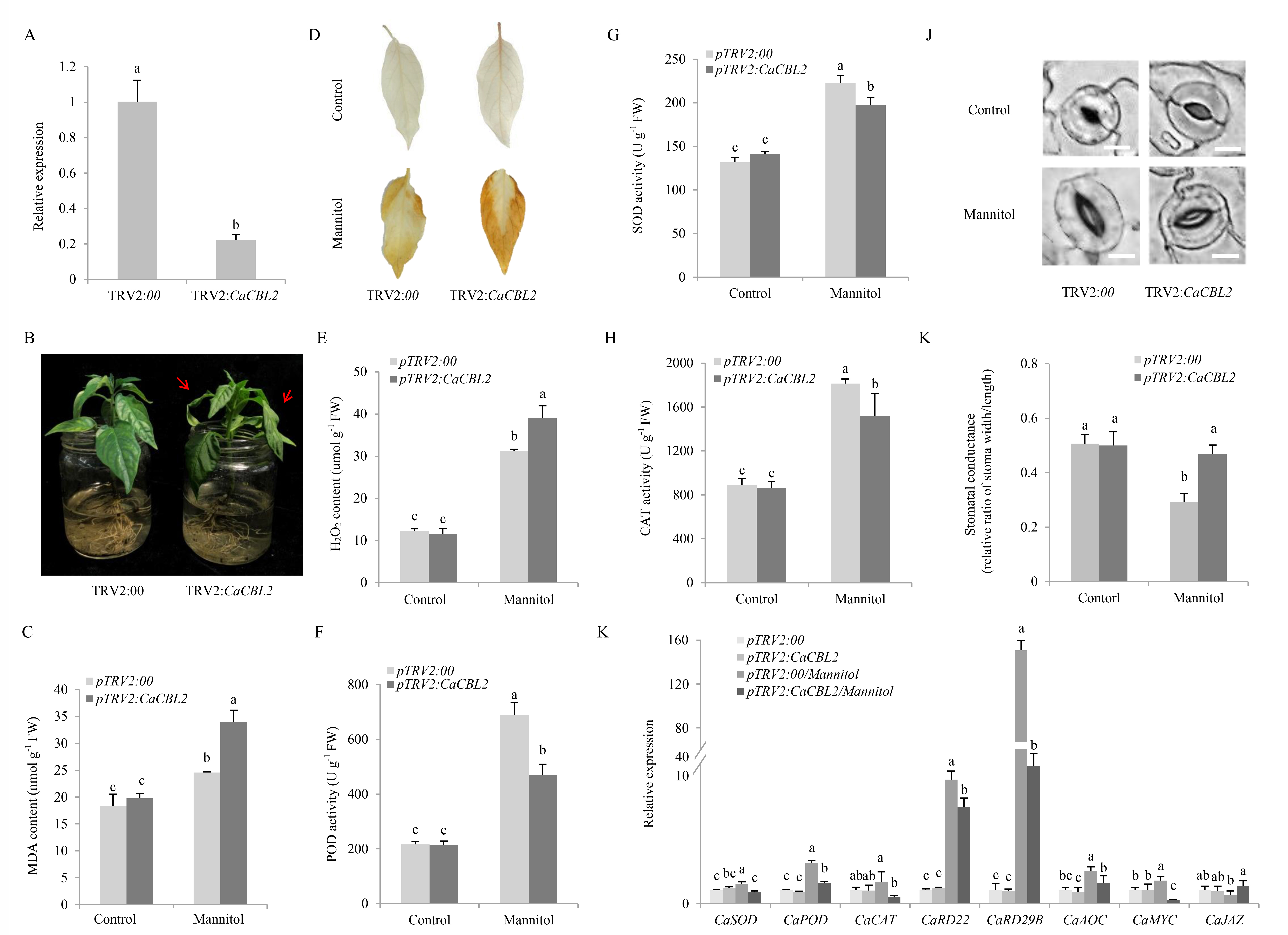

Supplement: Supplementary file 5 — Supplementary Fig. S4 [file 41438_2021_651_MOESM5_ESM.jpg]
